# Reversal of pentylenetetrazole-altered swimming and neural activity-regulated gene expression in zebrafish larvae by valproic acid and valerian extract

**DOI:** 10.1007/s00213-016-4304-z

**Published:** 2016-05-11

**Authors:** Bianca A. Torres-Hernández, Luis R. Colón, Coral Rosa-Falero, Aranza Torrado, Nahira Miscalichi, José G. Ortíz, Lorena González-Sepúlveda, Naydi Pérez-Ríos, Erick Suárez-Pérez, John N. Bradsher, Martine Behra

**Affiliations:** School of Medicine, Department of Pharmacology and Toxicology, Medical Science Campus of the University of Puerto Rico (MCS-UPR), San Juan, PR USA; School of Medicine, Department of Anatomy and Neurobiology, Medical Science Campus of the University of Puerto Rico (MCS-UPR), San Juan, PR USA; Puerto Rico Clinical and Translational Research Consortium (PRCTRC), Medical Science Campus of the University of Puerto Rico (MCS-UPR), San Juan, PR USA

**Keywords:** Crude extracts, In vivo high-throughput psychoactivity screening, Psychoactive plants, Zebrafish larva behavior, Valerian, Ethnobotany, *c-fos*, *npas4a*, *bdnf*

## Abstract

**Rationale:**

Ethnopharmacology has documented hundreds of psychoactive plants awaiting exploitation for drug discovery. A robust and inexpensive in vivo system allowing systematic screening would be critical to exploiting this knowledge.

**Objective:**

The objective of this study was to establish a cheap and accurate screening method which can be used for testing psychoactive efficacy of complex mixtures of unknown composition, like plant crude extracts.

**Methods:**

We used automated recording of zebrafish larval swimming behavior during light vs. dark periods which we reproducibly altered with an anxiogenic compound, pentylenetetrazole (PTZ). First, we reversed this PTZ-altered swimming by co-treatment with a well-defined synthetic anxiolytic drug, valproic acid (VPA). Next, we aimed at reversing it by adding crude root extracts of *Valeriana officinalis* (Val) from which VPA was originally derived. Finally, we assessed how expression of neural activity-regulated genes (*c-fos*, *npas4a*, and *bdnf*) known to be upregulated by PTZ treatment was affected in the presence of Val.

**Results:**

Both VPA and Val significantly reversed the PTZ-altered swimming behaviors. Noticeably, Val at higher doses was affecting swimming independently of the presence of PTZ. A strong regulation of all three neural-activity genes was observed in Val-treated larvae which fully supported the behavioral results.

**Conclusions:**

We demonstrated in a combined behavioral-molecular approach the strong psychoactivity of a natural extract of unknown composition made from *V. officinalis*. Our results highlight the efficacy and sensitivity of such an approach, therefore offering a novel in vivo screening system amenable to high-throughput testing of promising ethnobotanical candidates.

**Electronic supplementary material:**

The online version of this article (doi:10.1007/s00213-016-4304-z) contains supplementary material, which is available to authorized users.

## Introduction

Extensive ethnopharmaceutical documentation is available on plants with demonstrated psychoactivity in humans (for review (Raetsch 2005)). Even though there is a huge need for new molecules in mental health, this reservoir of knowledge remains greatly untapped. One of the main reasons is probably the lack of a good in vivo system allowing testing of psychoactive efficacy of complex mixtures of unknown composition. Ethnobotanical reports on a specific plant often differ on plant species/subspecies/harvest but also on mode of preparation and administration which all generate a number of imponderable parameters. Therefore, it is necessary to establish a system which would be capable of circumventing those problems. Ideally, the proposed system has to be flexible and sensitive enough to allow rapid and systematic screening of a big number of complex mixtures like crude plants extracts. In such a system, first pass screening could be done with minimally processed extracts like preparations made from dried powdered parts of a plant of interest. Subsequently, gradually refined mixtures could be tested the same way to eventually isolate the active compound(s).

Psychoactivity, unlike other physiologic activities, needs to be tested in an organism with an elaborate nervous system. Classical animal models like rodents are expensive, which is a major drawback for first-pass screening, and drug administration can be challenging. We used instead zebrafish larvae which have been successfully used for drug screening and are amenable to high throughput (Rennekamp and Peterson 2015). An adult couple can yield weekly several hundred eggs, which develop rapidly and externally (Kimmel et al. 1995). At 5 days post-fertilization (dpf), the fully developed little fish will exhibit a range of sophisticated behaviors like escaping predators and catching prey (Muto and Kawakami 2013). Drugs can be simply added to the water in which animals swim for the entire duration of the experiment. Uniform exposure and drug delivery from animal to animal can be assumed, even if there is still little known about pharmacokinetics of psychoactive compounds in zebrafish (MacRae and Peterson 2015).

We based our screening assay on a quantifiable, strong behavior: the response of fish to light and dark conditions which is already present in young larvae (MacPhail et al. 2009). Fish swim considerably less when exposed to strong light and significantly more when in the dark. After an abrupt light change, animals adjust activity by drastically decreasing swimming when the light is turned on and drastically increasing it when the light is turned off. It has been referred to as the photomotor response (PMR) (Kristofco et al. 2015). PMR or the short post-transition period after a light change is followed by a period of habituation which can be repeatedly interrupted by switching light conditions again, thus creating a highly predictable light-dependent cyclic behavior. Remarkably, this strong behavior can be profoundly modified by known anxiogenic/convulsive drugs like aconitine or pentylenetetrazole (PTZ) (Ellis et al. 2012; Ellis and Soanes 2012). We could reliably and reproducibly elicit swimming alterations over several light and dark cycles with a set PTZ concentration of 7.5 mM (PTZ_7.5_). PTZ_7.5_-altered swimming was maximal in post-transitions. (1) When turning the light on, we measured a huge increase in swimming or “hyperactivity” in opposition to the sharp decrease in activity seen in untreated control animals. (2) On the contrary, when turning the light off, we measured an immediate drastic decrease in activity resembling “freezing” in opposition to the stark increase in activity seen in untreated control animals. In addition to those two measures of anxiety, we assessed a third parameter: (3) light-dependent center avoidance. PTZ_7.5_ application elicited no center avoidance in light and provoked it in dark in complete opposition with the behavior of untreated control larvae which were avoiding the center in light conditions only. We hypothesized that those three defined parameters of the PTZ_7.5_-altered swimming could be used for measuring efficacy of a psychoactive compound which would be present in the water as a highly purified synthetic molecule or as a part of a complex mixture. An efficient molecule would reverse at least one or more of the three parameters of the PTZ_7.5_-altered swimming behavior in a concentration-dependent manner.

To validate this in vivo system, we tested aqueous crude extracts of a fairly well-studied psychoactive plant: *Valeriana officinalis*. Commonly called Valerian (Val), its root extracts are widely used as dietary supplement to treat insomnia and anxiety (Fugh-Berman and Cott 1999). Small clinical studies have yielded controversial results on its efficacy (Bent et al. 2006; Fernandez-San-Martin et al. 2010; Taibi et al. 2009). In vivo studies mostly agree on its anxiolytic (Murphy et al. 2010) and anti-convulsive (Rezvani et al. 2010) but not sedative (Hattesohl et al. 2008) properties. In vitro assays showed that crude extracts and isolated constituents like valerenic acid (Benke et al. 2009), alkaloids, and lignans can interact with GABA_A_ (Cavadas et al. 1995), glutamate (Del Valle-Mojica et al. 2011a), adenosine (Lacher et al. 2007), and serotonin (Dietz et al. 2005) receptors. This argues for synergistic action of the more than 150 biomedically relevant chemicals which have been isolated from Val crude extracts (Patočka 2010). More relevant to this study is another major constituent, valeric acid, which led to the synthesis of an analog, valproic acid (VPA), which is the primarily prescribed mood stabilizer for bipolar disorders (Chiu et al. 2013). We used VPA to validate our behavioral screening assay and established a dose-response profile with four different concentrations. We proceeded likewise with Val root crude extracts. Our results clearly showed a significant concentration-dependent reversal of all three assessed parameters of the PTZ_7.5_-altered swimming with both VPA and Val. In addition, with higher concentrations of VPA-alone treatments, we found light-independent hypoactivity. This was reminiscent of sedation induced by higher dosage that has been extensively documented in animals (Berghmans et al. 2007) and humans (Chateauvieux et al. 2010), thus validating the relevance of the usage of this lower vertebrate for psychoactivity testing. Furthermore, we found that Val, at all concentrations tested, consistently elicited hyperactivity in a PTZ-independent manner, which might be accounted for by one or more of the multiple active compounds potentially present in the complex mixture that we tested.

To confirm our behavioral results with Val extracts, we tracked changes in neuronal activity in the brain of treated larvae. To do so, we analyzed the gene expression patterns of a small set of neural activity upregulated genes. Those genes were previously shown to be transcriptionally upregulated in response to increased brain synaptic activity in general and to PTZ treatments in particular (Baxendale et al. 2012). We chose *c-fos*, the gold standard for detecting synaptic activity (Zhang et al. 2002); *npas4a*, involved in regulating excitatory-inhibitory balance within neural circuits (Bloodgood et al. 2013; Lin et al. 2008; Spiegel et al. 2014); and the neurotrophic factor *bdnf*, a putative downstream target of both *c-fos* and *npas4a* (Greenberg et al. 2009; Lin et al. 2008). We analyzed how the different treatments affected the transcription of all three genes, first by whole-mount in situ hybridization (WISH) to establish changes in expression domain in larval brains and second by qPCRs to quantify the changes. We showed strong transcriptional down-regulation of synaptic activity genes in animals co-treated with Val and PTZ_7.5_ for all three genes. We also found the transcription of *c-fos* to be upregulated in larvae treated with Val-alone when comparing to untreated control animals. Therefore, our molecular data fully corroborated and consolidated our behavior results. In addition, we showed for the first time that Val strongly regulates transcription of neuronal activity upregulated genes in the brain, thus providing the first molecular evidence of the psychoactive efficacy of a complex mixture minimally prepared from plant crude extracts.

Taken together, our coupled behavioral-molecular approach demonstrated the sensitivity of our in vivo system in detecting psychoactivity, even in a complex mixture of unknown composition, namely, an aqueous plant crude extract. Therefore, we are providing a means for systematic and high-throughput testing of strong ethnobotanical psychoactive candidates in the quest of new psychiatric drugs.

## Materials and methods

### Fish strain/care/husbandry

For breeding, we used an original strain of zebrafish (*Danio rerio*) known as “TAB-5” made from a hybrid cross between fish from two of the most commonly used zebrafish lines, namely, Tübingen, TUB, (Haffter et al. 1996) and AB (Streisinger et al. 1981). TAB-5 was first isolated in 1997 and maintained without ever introducing any other outside genetic diversity. At least once a year, pools of siblings were crossed and their offspring raised to generate the next generation. A strong mating pair was used to derive the NHGR-1 line which has been sequenced to a depth of ∼50×, and all polymorphisms have been mapped (LaFave et al. 2014). All of our husbandry protocols are in accordance with standard procedures (Westerfield 1993) and the IACUC-authorized protocol (no. A880213). Fish were raised at 28 °C on 14:10-h light/dark cycles on a recirculating system (Techniplast®). Water supplied to the system was filtered by reverse osmosis (Siemens) and maintained at a neutral pH (∼7.0–7.5) and stable conductivity (∼1000 μS/cm) by adding Instant Ocean® sea salts. We refer to it herein as system water (SW). Fertilized embryos were raised in SW at 28 °C on 14:10-h light/dark cycles, and larvae with inflated swim bladders (that we used as an internal readout for good health) and no obvious abnormalities were selected for subsequent experiments.

### Chemicals

PTZ and VPA were obtained from Sigma-Aldrich. Stock solutions were prepared in SW and stored at 4 °C for less than 2 weeks. We tested serial dilutions of PTZ ranging from 2 to 10 mM and empirically determined that PTZ_7.5_ was optimal for triggering a reproducible PTZ-altered swimming in larvae. We used PTZ_7.5_ in all subsequent experiments. We established a dose-response profile for VPA with four concentrations (VPA = 0.5, 1, 2, and 3 mM) based on previous work done in zebrafish (Berghmans et al. 2007; Ellis and Soanes 2012). We established survival curves for both PTZ_7.5_ and VPA for all the above mentioned concentrations, and we did not see significant effects for at least 24 h, which exceeded by far the timeline of our assay (data not shown), thus validating the assay for freedom from toxic influence of the test system.

### Valerian root extract preparation

We exclusively prepared aqueous plant extracts to avoid ethanol residues, which could affect the animals and confound results (Parker et al. 2014). Preparations were always made ad hoc to avoid degradation of the solutions. We purchased (from Pacific Botanicals, Grants Pass, OR) dry powdered valerian roots certified organically grown and minimally processed as described by the seller (www.pacificbotanicals.com). We acquired a large enough quantity to perform all needed experiments in order to avoid possible harvest-to-harvest variations which have been described previously (Patočka 2010). The dry powder was kept in a light-opaque tightly sealed container at +4 °C and handled with extreme care to avoid contamination. We prepared the extracts following a previously published method (Del Valle-Mojica et al. 2011b; Torres-Hernández et al. 2015). In brief, we prepared ad hoc a stock solution (=100 mg/ml) of dry powdered valerian roots which we magnetically stirred into SW in a covered beaker at RT (25 °C) for 1 h. Next, we filtered the solution through coarse paper to remove the residual suspensions and to homogenize the extract. We diluted further with SW to the desired concentration. To establish a dose-response profile for Val, we used previously reported work in zebrafish adults (Torres-Hernández et al. 2015). We tested four concentrations (Val = 1, 2.5, 5, and 7 mg/ml). We also established survival curves with all of those concentrations, and we did not see significant effects for at least 24 h which exceeded by far the timeline of our assay (data not shown), thus again validating the assay for freedom from toxic influence of the test system.

### Dark/light response behavioral assay in larvae

#### Plate setup and drug administration

For each behavioral experiment, we recorded 48 animals in parallel (= one 48-well plate (CELLSTAR®) with one larva/well) and performed independent triplicates for all treatments. Preliminary testing confirmed that there was no bias induced by larvae occupying a specific location in the plate (data not shown), thus freeing us from having to randomize fish location according to treatment designation throughout experiments. We collected eggs from group spawning (∼5–7 females with 8–10 males) of wild-type animals (TAB-5). We raised embryos in clean SW that we replaced daily and maintained them in a light-cycled incubator (14:10 h light/dark) set at 28 °C. To allow animals to adapt to the new environment, we plated one single healthy 5-dpf larva in each well in 450 μl SW 1 day prior to recording. Next day, we checked the health of the animals before topping off wells to 500 μl with SW or SW with drugs (PTZ, VPA, or Val) to the desired concentration. When determining the optimal PTZ concentration to induce a reproducible altered swimming, we split the plates in two with the three top rows containing untreated larvae (Unt, *n* = 3 × 8 = 24) and the three bottom rows containing larvae treated with the PTZ concentration under study (PTZ, *n* = 24). We averaged swimming distances per larva from the triplicate independent experiments (Unt, *n* = 72, and PTZ, *n* = 72).

For each tested concentration of VPA and Val, we systematically measured in parallel the swimming activity of (1) untreated larvae, (2) PTZ_7.5_-alone-treated larvae, (3) compound-alone-treated larvae, and (4) PTZ_7.5_ + tested compound-treated larvae. We averaged the results from the triplicate independent experiments and reported swimming distance/min/larva for each treatment as exemplified for Val 1 mg/ml = Val_1_ (Unt *n* = 24; PTZ_7.5_*n* = 24; Val_1_*n* = 48; and PTZ_7.5_ + Val_1_*n* = 48). Once we added the drugs, we immediately put the plate into the ZebraBox (Viewpoint, France) and started the recording (∼3 min). For the first 27 min, larvae were in the dark to adapt to the light condition. Next, we submitted larvae to four successive cycles of alternating 10 min of bright light (L) and complete dark (D).

#### Swimming activity tracking

We empirically partitioned the recording and reporting of larval activity according to three swim speeds: slow (S1), sustained (S2), and fast (S3), which we determined based on prior reports (Ellis et al. 2012), preliminary swim speed analyses, and manufacturer’s recommendation. Slow swim speeds (S1 <0.2 cm/s) were reporting minimal activity of larva which was at the limit of detection by the recording device. Fast swim speeds (S3 >2 cm/s) were relatively rarely seen, always short-lasting, and corresponded to intermittent powerful larval accelerations. Sustained swim speeds (0.2 cm/s < S2 < 2 cm/s) were observed during the remainder of larval activity. We further partitioned the recording of swimming activity according to the location in the well (total well diameter, d_o_ = 1 cm). A virtual inner open space was delimited (inner circle, d_i_ = 0.5 cm), and traveled distances were designated as total distances when considering the entire well as opposed to inner distances when considering solely the inner open space.

### Statistical analyses

#### Behavioral assay statistical analyses

**Fig. 1:**
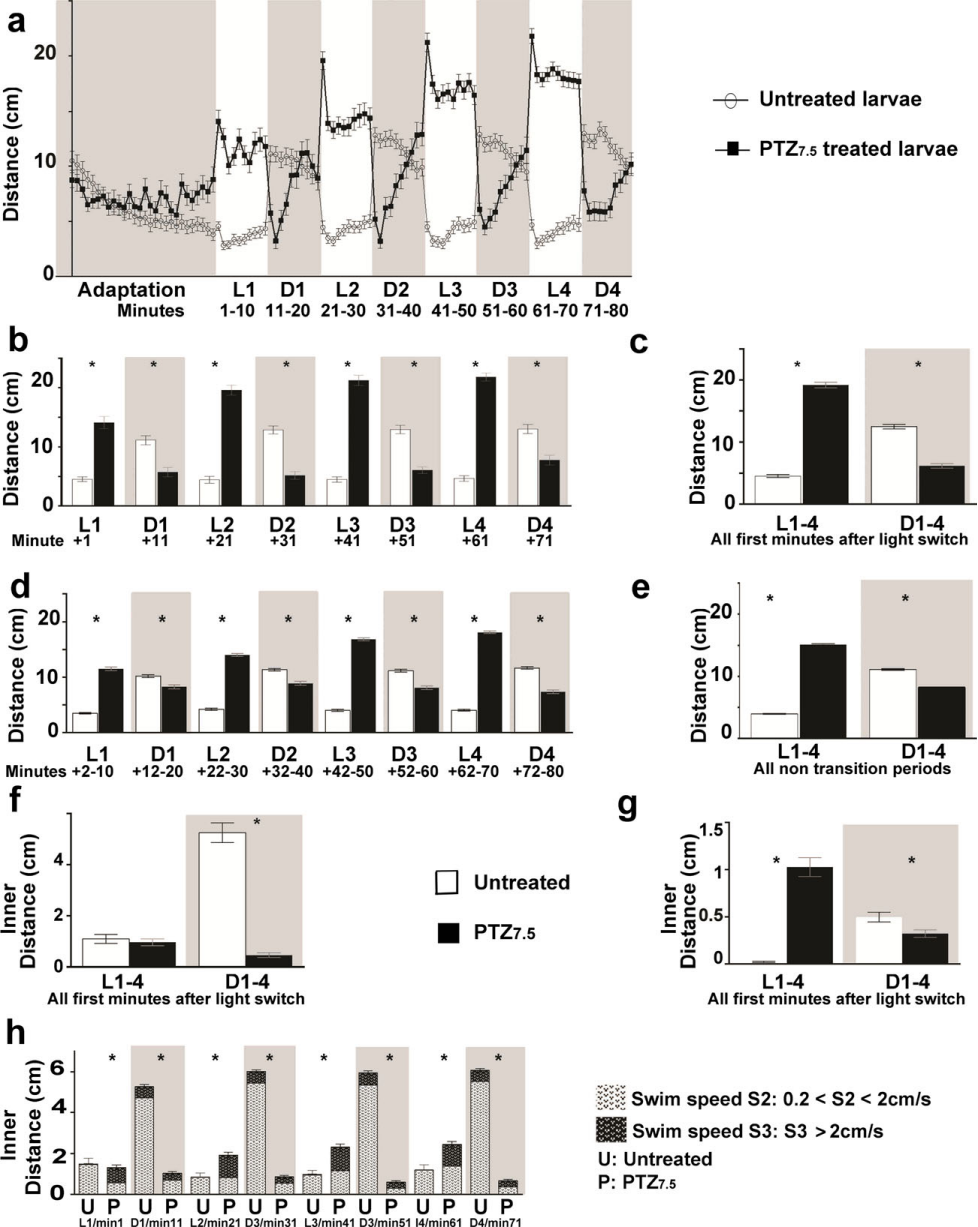
PTZ_7.5_ alters light-dependent swimming behaviors in zebrafish larvae. **a** Mean traveled distances (cm) by untreated () and PTZ_7.5_-treated () larvae reported minute by minute for the entire 80 min of recording. After an adaptation period to dark (*gray box*), larvae were submitted to four successive 10-min alternating light (*L1–L4*: *white zones*) and dark periods (*D1–D4*: *gray zones*). **b** Distances traveled in all successive post-transitions (=first minutes after a light switch) by untreated () and PTZ_7.5_-treated larvae (). **c** Distances traveled in cumulative post-transitions in light (*L 1–4*, *white boxes*) and in dark *(D 1–4*, *grey boxes*). **d** Distances traveled during all successive non-transitions (= remaining 9 min of each light or dark periods). **e** Distances traveled in compiled non-transitions. **f** Inner distances traveled in swim speed S2 (0.2 cm/s < S2 < 2 cm/s) in compiled post-transitions. **g** Inner distances traveled in swim speed S3 (>2 cm/s) in compiled post-transitions. **h** Inner distances traveled in all successive post-transitions in swim speed S2 () and S3 (). *Error bars* representing SEM. *p values <0.001 (detailed values are presented in Supplementary Table 1)

**Fig. 2:**
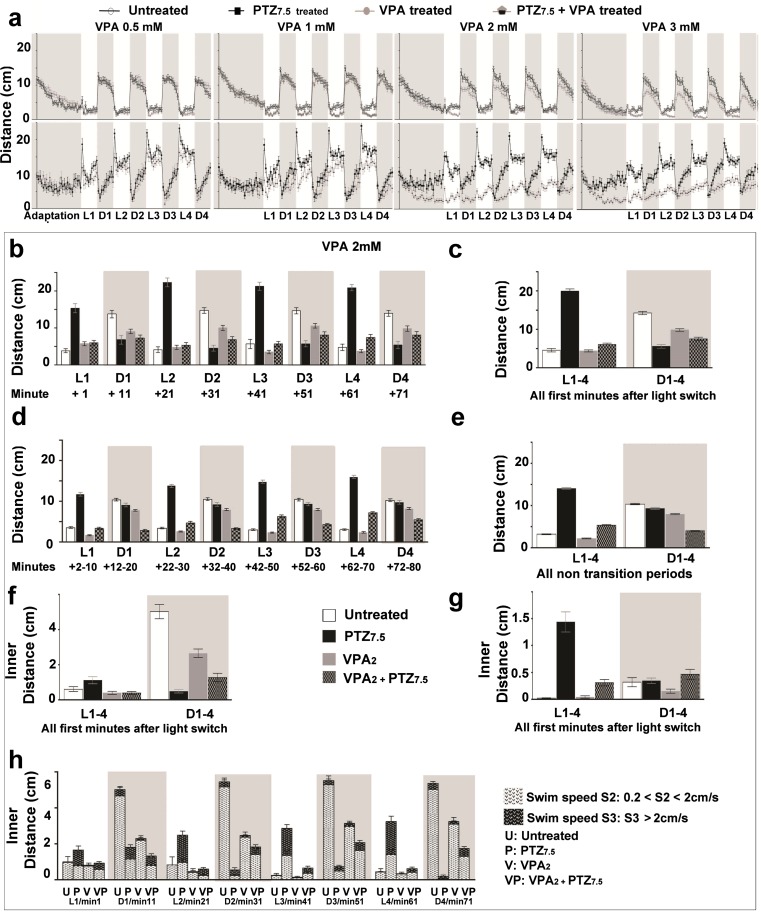
Valproate reverses PTZ_7.5_-altered light-dependent swimming behavior. **a** Mean traveled distances (cm) by untreated () and VPA-alone-treated () larvae in *top panels*, and by PTZ_7.5_-alone-treated () and VPA + PTZ_7.5_-co-treated () larvae in *bottom panels*. Larvae were treated with increasing VPA concentrations: 0.5 mM (VPA_0.5_, *left column*), 1 mM (VPA_1_, *second column*), 2 mM (VPA_2_, *third column*), and 3 mM (VPA_3_, *fourth column*). **b** Distances traveled during all successive post-transitions by untreated (), PTZ_7.5_-alone-treated (), VPA_2_ alone (), and PTZ_7.5_ + VPA_2_-co-treated larvae (). **c** Distances traveled during cumulative post-transitions. **d** Distances traveled during all successive non-transitions (= remaining 9 min of each light or dark periods). **e** Distances traveled during compiled non-transitions. **f** Inner distances traveled in swim speed S2 (0.2 cm/s < S2 < 2 cm/s) during compiled post-transitions. **g** Inner distances traveled in swim speed S3 (>2 cm/s) during compiled post-transitions. **h** Inner distances traveled during all compiled post-transitions in swim speed S2 () and S3 () by untreated (*U*), PTZ_7.5_-alone-treated (*P*), VPA_2_-alone-treated (*V*), and VPA_2_ + PTZ_7.5_-co-treated (*VP*) larvae. *Error bars* represent SEM. *Symbols* for significant *p* values were omitted for clarity but are discussed in the “Results” section and detailed in Supplementary Tables 2, 3, 4, and 5

**Fig. 3:**
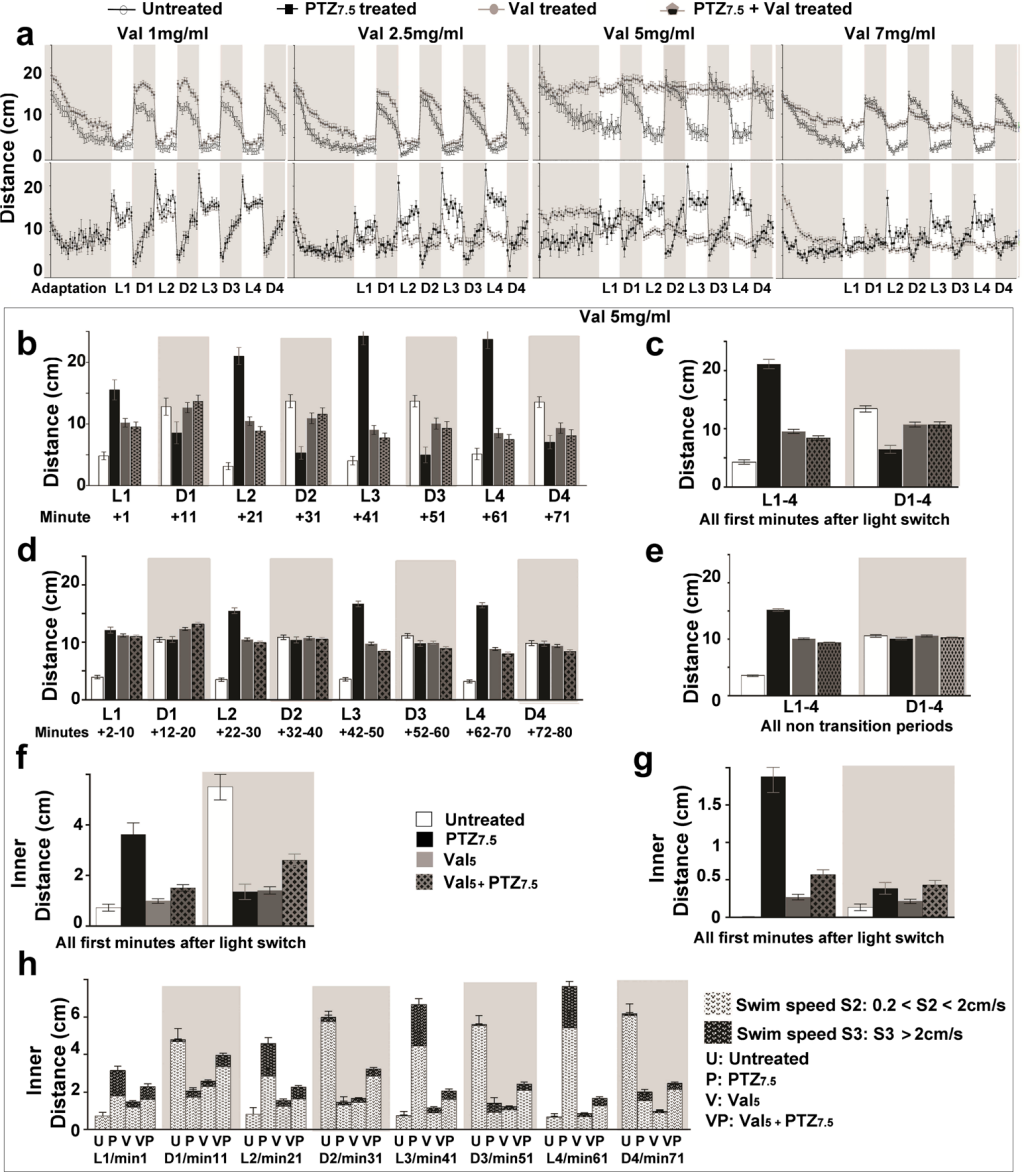
Valerian extract (Val) reverses the PTZ_7.5_-altered light-dependent swimming behavior. **a** Mean traveled distances (cm) by untreated () and Val-alone-treated () larvae in *top panels*, and by PTZ_7.5_-alone-treated () and Val + PTZ_7.5_-co-treated () larvae in *bottom panels*. Larvae were treated with increasing Val concentrations: 1 mg/ml (Val_1_, *left column*), 2.5 mg/ml (Val_2.5_, *second column*), 5 mg/ml (Val_5_, *third column*), and 7 mg/ml (Val_7_, *fourth column*). **b** Distances traveled during all successive post-transitions by untreated (), PTZ_7.5_-alone-treated (), Val_5_ alone (), and Val_5_ + PTZ_7.5_-co-treated larvae (). **c** Distances traveled during compiled post-transitions. **d** Distances traveled during all compiled non-transitions. **e** Distances traveled during compiled non-transitions. **f** Inner distances traveled in swim speed S2 (0.20 cm/s < S2 < 2 cm/s) during compiled post-transitions. **g** Inner distances traveled in swim speed S3 (>2 cm/s) during cumulative post-transitions. **h** Inner distances traveled during all successive post-transitions in swim speed S2 () and S3 () by untreated (*U*), PTZ_7.5_-alone-treated (*P*), Val_5_-alone-treated (*V*), and Val_5_ + PTZ_7.5_-co-treated (*VP*) larvae. *Error bars* represent SEM. *Symbols* for significant p values were omitted for clarity but are discussed in the “Results” section and detailed in Supplementary Tables 6, 7, 8, and 9

We analyzed averaged total distances traveled per larva from the triplicate experiments using GraphPad Prism (v.6) and plotted them vs. a time axis, displaying the entire time-course of the experiment. We included in all graphs the error bars which represent the mean ± standard error of the mean (SEM). Further statistical analyses were performed using the software Stata (v.14). We carried out a first set of parametric analyses using the mixed command in Stata. We analyzed total traveled distances in two swim speeds (S2 and S3) for all VPA and Val concentrations tested segregated by light patterns. First, we analyzed the compiled distances traveled during the entire 10 min of all dark periods (D = D1 + D2 + D3 + D4) or all light periods (L = L1 + L2 + L3 + L4). Next, we distinguished post-transitions (= first minute after a light change), from all four light to dark (L/D) and all four dark to light (D/L) changes. And, finally, we distinguished all compiled non-transitions in light or in dark periods (= remaining 9 min of all light or all dark periods, respectively). We analyzed the same way inner distances but only for post-transitions. For this first set of analyses, we used a linear regression model with categorical predictors with random intercept. The parameter estimation was performed using the multilevel linear regression model approach (Leyland 2004). This model allowed us to control the effect of the correlation among the number of fixed repeated measures on the same larvae. Based on this model, we made an assessment of the interaction between treatment and light pattern by performing six different comparisons at different time points: (1) untreated (Unt) vs. PTZ_7.5_ (PTZ); (2) Unt vs. VPA and Unt vs. Val; (3) Unt vs. PTZ + VPA and Unt vs. PTZ + Val; (4) PTZ vs. VPA and PTZ vs. Val; (5) PTZ vs. PTZ + VPA and PTZ vs. PTZ + Val; and (6) VPA vs. PTZ + VPA and Val vs. PTZ + Val. Selected results are discussed in the “Results” section and summarized in Supplementary Tables (Table 1 for PTZ treatments corresponding to Fig. 1, Table 2 for VPA treatments corresponding to Fig. 2, and Table 6 for Val treatments corresponding to Fig. 3).

In order to analyze individual transitions for each treatment, we performed a second set of parametric analyses. Due to the heterogeneity of variances found after the Bartlett’s tests, we performed modified Brown and Forsythe tests which are several *t* tests with unequal variances which we performed for each transition and for each treatment. To control the experiment-wise type I error that resulted from those multiple *t* tests, we used the *F* Fisher probability distribution as recommended by Tamhane and Bechhofer (Tamhane and Bechhofer 1979). Selected results are discussed in the “Results” section and summarized in Supplementary Tables (Tables 3, 4, and 5 for VPA corresponding to Fig. 2 and Tables 7, 8, and 9 for Val corresponding to Fig. 3). All statistical results from the separate analyses were consistent throughout experiments. Complete detailed results are available upon request.

#### qPCR data statistical analysis

After each run, ΔC_t_ was determined with the EcoStudy Software (Illumina) using two reference genes and normalized against the untreated sample. Fold change was calculated using the 2^−ΔΔC^_t_ method (Livak and Schmittgen 2001), and level of expression in untreated animals was defined as 1. Results were compiled using GraphPad Prism. Errors bars represented the 95 % confidence interval, and statistical difference was tested with the non-parametric Kruskal-Wallis test.

### Whole-mount in situ hybridization

WISH was performed as described (Thisse and Thisse 2008). All antisense probes were prepared from plasmids kindly gifted by Dr. Vincent Cunliffe (Baxendale et al. 2012). Imaging was performed with an inverted Axiovert (Zeiss) equipped with AxioCam. Acquisitions were done using the AxioVision software. Final figures were assembled in Photoshop. The total number of WISH-stained animals for each antisense probe were as follows: *c-fos* (Unt *n* = 22, PTZ_7.5_*n* = 23, Val_5_*n* = 42, PTZ_7.5_ + Val_5_*n* = 29); *bdnf* (Unt *n* = 23, PTZ_7.5_*n* = 33, Val _5_*n* = 29, PTZ_7.5_ + Val_5_*n* = 30); *npas4a* (Unt *n* = 24, PTZ_7.5_*n* = 30, Val_5_*n* = 32, PTZ_7.5_ + Val_5_*n* = 32); *gbr2* (Unt *n* = 20, PTZ_7.5_*n* = 31, Val_5_*n* = 39, PTZ_7.5_ + Val_5_*n* = 18); and *gabra1* (Unt *n* = 27, PTZ_7.5_*n* = 28, Val_5_*n* = 34, PTZ_7.5_ + Val_5_*n* = 30).

### Quantitative PCRs

Total RNA was extracted from pools of whole larvae (6 dpf, *n* ∼50) using RNeasy® Plus Universal Mini Kit (Qiagen) following manufacturer’s protocol. Three biological samples/treatment were made from three independent behavioral assays. RNA concentration was determined (NanoPhotometer P-Class, Implen) and cDNAs synthesized with 2 μg of RNA with SuperScript II Reverse Transcriptase (Invitrogen). Forward (F-) and reverse (R-) primers were as follows: *beta-actin* (*β-actin*) F-CATCCATCGTTCACAGGAAGTG and R-TGGTCGTTCGTTTGAATCTCAT; *elongation factor 1 alpha subunit (ef1a)* F-CTGCCAGTGTTGCCTTCGT and R-CCTTGCGCTCAATCTTCCA; *brain-derived neurotrophic factor (bdnf)* F-TCGAAGGACGTTGACCTGTATG and R-TGGCGGCATCCAGGTAGT, *peptide YYa* (*Pyya*) F-TCCTCATCTGCGTGCTTCTGT and R-GCGGTGTAATATTTGGCGAGTT; *GABAa receptor alpha1 (gabra1)* F-TCAGGCAGAGCTGGAAGGAT and R-TGCCGTTGTGGAAGAACGT; *c-fos* F-AACTGTCACGGCGATCTCTT and R-GCAGGCATGTATGGTTCAGA; and *npas4a* F-ATGGGTCTGGTTTACATGG and R-CTTGTCTGGGTTGAGAGGAAC. Final primer concentrations were determined experimentally: 0.1 μM for *bdnf*; 0.3 μM for *pyya*, *gabra1*, and *gbrg2*; and 0.5 μM for *c-fos* and *npas4a*. Each gene along with two reference genes (*β-actin* 0.1 μM and *ef1a* 0.1 μM) was evaluated in parallel runs in an Eco™ Real-Time PCR machine (Illumina). Technical triplicates with three biological samples of all the different treatments were evaluated for each gene.

## Results

### PTZ_7.5_ alters light-dependent swimming behavior

We first assessed the larval light-dependent swimming activity and aimed at reproducibly altering it with PTZ application. To do so, we set up 48-well plates with one animal/well and treated half of them with a set concentration of PTZ, while leaving the other half untreated. Immediately after PTZ addition, we placed animals in the dark inside the recording device to measure the traveled distance/minute/larva in 48 parallel recordings. After an additional 27 min, we submitted larvae to 4 cycles of alternating light and dark periods of 10 min each. We divided the recording of all swimming activity according to three experimentally determined swim speeds (S1, S2, and S3). We calculated and represented means for the entire duration of the experiment. A highly reproducible light-dependent swimming pattern emerged for all swim speeds (S2 shown in Fig. 1a, S1 and S3 not shown). Untreated larvae () displayed low activity when exposed to light (L1 to L4 white boxes, Supplementary Table 1, Unt = 4.02 cm/min) which was nearly tripled when they were exposed to dark (D1 to D4 gray boxes, Unt = 11.25 cm/min). All tested PTZ concentrations altered significantly the swimming behavior (data not shown), but with PTZ 7.5 mM (=PTZ_7.5_), we could consistently trigger a quasi-opposite light-dependent swimming pattern. PTZ_7.5_-treated larvae (Fig. 1a and Supplementary Table 1) displayed an overall high activity when exposed to light (PTZ = 15.52 cm/min) which was lowered by half when they were exposed to dark (PTZ = 7.96 cm/min). Differences were highly significant in light and dark periods. Furthermore, differences were maximal during the first minute immediately following a light change in all successive post-transitions (Fig. 1b). In cumulative post-transitions (Fig. 1c) during light periods, untreated larvae traveled 4.54 cm/min vs. 19.17 cm/min, (*p* < 0.001) for PTZ_7.5_-treated larvae which were exhibiting strong hyperactivity. On the contrary, during dark periods, PTZ_7.5_-treated animals were freezing instead of increasing activity like untreated larvae (Unt = 12.47 cm/min vs. PTZ = 6.19 cm/min, *p* < 0.001). This remained true in non-transitions (Fig. 1e) with smaller but still highly significant differences during light periods (Unt = 3.96 cm/min vs. PTZ = 15.12 cm/min) and during dark periods (Unt = 11.11 cm/min vs. PTZ = 8.16 cm/min). Thus, with PTZ_7.5_, we profoundly altered the light-dependent swimming activity and elicited an opposite response to change in light conditions as compared to untreated controls.

In addition, we measured a well-accepted behavioral criterion for anxiety: avoidance of open space. To do so, we measured inner-traveled distances during post-transitions only as larvae were traveling very little to the center during non-transitions (data not shown). We found a clear light-dependent center avoidance in untreated larvae which was significantly altered by PTZ_7.5_ treatments in a least one of the swim speeds (S2 shown in Fig. 1f; S3 shown in Fig. 1g; cumulative S2 and S3 shown in Fig. 1h; S1 not shown). During light periods, untreated larvae were avoiding the center in both swim speeds, but PTZ_7.5_-treated animals did not in S3 (compare in Fig. 1g, Unt = 0.02 cm/min vs. PTZ = 1.03 cm/min, *p* < 0.001). When exposed to dark, in both S2 and S3 and in all post-transitions (Fig. 1h S2 and S3 cumulative), untreated animals increased activity in the center, as opposed to PTZ_7.5_-treated animals which decreased it. Differences were maximal in the sustained swim speed S2 (Fig. 1f, Unt = 5.18 cm/min vs. PTZ = 0.46 cm/min, *p* < 0.001) but still highly significant in the fast swim speed S3 (Fig. 1g, Unt = 0.57 cm/min vs. PTZ = 0.32 cm/min, *p* < 0.001). Thus, PTZ_7.5_ treatments were also clearly altering the light-dependent center avoidance. Unlike untreated larvae that were only traveling to the center in dark periods, PTZ_7.5_-treated larvae were only traveling to the center in light periods.

## Valproate reverses PTZ_7.5_-altered light-dependent swimming behavior

Next, we aimed at validating the use of this PTZ_7.5_-altered light-dependent swimming behavior in zebrafish larva and proving its relevance for humans. To do so, we tested a well-described psychoactive synthetic molecule, valproic acid (valproate = VPA). First, we established a dose-response profile for VPA and successively tested four concentrations (VPA_0_ = 0.5 mM, VPA_1_ = 1 mM, VPA_2_ = 2 mM, and VPA_3_ = 3 mM). For each VPA concentration, we recorded independent triplicate experiments of 48 larvae/plate set up as follows: 8 untreated, 8 PTZ_7.5_-alone-treated, 16 VPA-alone-treated, and 16 PTZ_7.5_ + VPA-co-treated larvae. We plotted the means against time for each concentration (Fig. 2a, VPA_0.5_ left panels, VPA_1_ center left panels, VPA_2_ center right panels, and VPA_3_ right panels). For clarity, we presented treatments pairwise: untreated with VPA alone (top row,  and , respectively) and PTZ_7.5_ alone with PTZ_7.5_ + VPA (bottom row,  and , respectively). Across all concentrations of VPA, in light periods, overall distances traveled by untreated vs. VPA-alone-treated animals were not significantly different (Fig. 2a top panels, white boxes). However, in dark periods, animals treated with the two higher concentrations traveled less (gray boxes and Supplementary Table 2: Unt = 10.75 cm/min vs. VPA_2_ = 8.15 cm/min, *p* < 0.001; Unt = 9.02 cm/min vs. VPA_3_ = 5.63 cm/min, *p* < 0.001), suggesting a concentration-dependent sedation.

Overall traveled distances by PTZ_7.5_-alone-treated larvae compared with distances traveled by PTZ_7.5_ + VPA-co-treated larvae were strongly reduced in all co-treatments and in a VPA concentration-dependent manner in light periods (PTZ_7.5_ = 15.13 cm/min vs. PTZ_7.5_ + VPA_0.5_ = 12.53 cm/min, *p* < 0.002; PTZ_7.5_ = 15.12 cm/min vs. PTZ_7.5_ + VPA_1_ = 10.39 cm/min, *p* < 0.001; PTZ_7.5_ = 14.65 cm/min vs. PTZ_7.5_ + VPA_2_ = 5.44 cm/min, *p* < 0.001; PTZ_7.5_ = 13.61 cm/min vs. PTZ_7.5_ + VPA_3_ = 6.07 cm/min, *p* < 0.001), suggesting reversal of the hyperactivity. This was even more striking in post-transitions. Hyperactivity in light was reversed in all co-treatments and was reduced by nearly 3-fold with the two higher VPA concentrations (PTZ_7.5_ = 21.46 cm/min vs. PTZ_7.5_ + VPA_0.5_ = 14.82 cm/min, *p* < 0.001; PTZ_7.5_ = 21.90 cm/min vs. PTZ_7.5_ + VPA_1_ = 10.95 cm/min, *p* < 0.001; PTZ_7.5_ = 19.95 cm/min vs. PTZ_7.5_ + VPA_2_ = 6.96 cm/min, *p* < 0.001; PTZ_7.5_ = 17.21 cm/min vs. PTZ_7.5_ + VPA_3_ = 6.19 cm/min, *p* < 0.001). Freezing observed in post-transitions in dark periods was corrected in co-treatments with VPA_2_ (Fig. 2c, Table 2, PTZ_7.5_ = 5.61 vs. PTZ_7.5_ + VPA_2_ = 7.55 cm/min, *p* < 0.001), and this was true in all successive time periods (Fig. 2b and Supplementary Table 3).

We analyzed the third parameter, light-dependent center avoidance in post-transitions for VPA_2_ concentration. In light periods, the lack of center avoidance by PTZ_7.5_-alone-treated larvae was reversed in PTZ_7.5_ + VPA_2_-co-treated larvae in both swim speeds: S2 (Fig. 2f, PTZ_7.5_ = 1.12 cm/min vs. PTZ_7.5_ + VPA_2_ = 0.39 cm/min, *p* < 0.001); and S3 (Fig. 2g, PTZ_7.5_ = 1.44 cm/min vs. PTZ_7.5_ + VPA_2_ = 0.31 cm/min, *p* < 0.001). In dark periods, center avoidance seen only in PTZ_7.5_-alone-treated larvae was also significantly reversed in co-treated larvae in swim speed, S2 (Fig. 2f, PTZ_7.5_ = 0.48 cm/min vs. PTZ_7.5_ + VPA_2_ = 1.27 cm/min, *p* < 0.001). Thus, PTZ-altered light-dependent center avoidance was significantly reversed in at least one swim speed in all successive post-transitions in light and in dark periods (Fig. 2h, S2 and S3 compiled for all successive post-transitions).

## *Valeriana officinalis* reverses PTZ_7.5_-altered light-dependent swimming behavior

Next, we wanted to see how a complex mixture like a plant crude extract of unknown composition could reverse PTZ_7.5_-altered swimming. To establish a dose-response profile, we treated larvae with four concentrations of aqueous valerian root extracts (Val 1 mg/ml = Val_1_, Val 2.5 mg/ml = Val_2.5_, Val 5 mg/ml = Val_5_, and Val 7 mg/ml = Val_7_). We noticed a number of features not observed previously with the VPA treatments. In light conditions, across all Val concentrations, Val-alone-treated animals (Fig. 3a top panels, ) were always traveling more than untreated larvae () suggesting moderate hyperactivity (Supplementary Table 6: Unt = 2.88 cm/min vs. Val_1_ = 4.59 cm/min, *p* = 0.05; Unt = 2.62 cm/min vs. Val_2.5_ = 4.29 cm/min, *p* = 0.044; Unt = 3.60 cm/min vs. Val_5_ = 10.00 cm/min, *p* < 0.001; Unt = 2.61 cm/min vs. Val_7_ = 6.33 cm/min, *p* < 0.001). Interestingly in dark conditions, this hyperactivity was decreasing in a concentration-dependent manner (Unt = 10.24 cm/min vs. Val_1_ = 15.09 cm/min, *p* < 0.001; Unt = 9.88 cm/min vs. Val_2.5_ = 13.31 cm/min, *p* < 0.001; Unt = 10.86 cm/min vs. Val_5_ = 10.58 cm/min, *p* = 0.788; Unt = 10.67 cm/min vs. Val_7_ = 8.38 cm/min, *p* < 0.001). Noticeably, animals treated with Val_5_ and Val_7_ seemed to only mildly adjust distances in response to light and dark changes. Furthermore, in light periods, the swimming activity of Val-alone-treated larvae vs. activity of co-treated PTZ_7.5_ + Val larvae was only significantly different in the two lower Val concentrations (Val_1_ = 4.59 cm/min vs. PTZ_7.5_ + Val_1_ = 15.09 cm/min, *p* < 0.001; Val_2.5_ = 4.29 cm/min vs. PTZ_7.5_ + Val_2.5_ = 9.00 cm/min, *p* < 0.001). The same was true in dark periods (Val_1_ = 15.09 cm/min vs. PTZ_7.5_ + Val_1_ = 9.04 cm/min, *p* < 0.001; Val_2.5_ = 13.31 cm/min vs. PTZ_7.5_ + Val_2.5_ = 7.66 cm/min, *p* < 0.001). Remarkably, for the two higher concentrations, distances traveled by Val alone and PTZ + Val-co-treated larvae were similar.

Next, we compared overall distances traveled by PTZ_7.5_-alone-treated larvae (Fig. 3a bottom panels, ) with overall distances traveled by PTZ_7.5_ + Val-co-treated larvae (). In light periods, we observed a Val concentration-dependent reduction of the PTZ_7.5_-induced hyperactivity starting with Val_2.5_ co-treatments (PTZ_7.5_ = 14.55 cm/min vs. PTZ_7.5_ + Val_2.5_ = 9.00 cm/min, *p* < 0.001; PTZ_7.5_ = 15.77 cm/min vs. PTZ_7.5_ + Val_5_ = 9.25 cm/min, *p* < 0.001; PTZ_7.5_ = 10.30 cm/min vs. PTZ_7.5_ + Val_7_ = 5.98 cm/min, *p* < 0.001). Remarkably in dark periods, averaged overall distances were not significantly different. However, when observing the behavior minute by minute, it was clearly different also starting with Val_2.5_ co-treatments (compare  and larvae  lines in Fig. 3a bottom panels, gray boxes). PTZ_7.5_-treated animals were gradually increasing distances from ∼2 cm/min to ∼15 cm/min while co-treated animals were steadily traveling ∼8 cm/min.

In post-transitions, the strong hyperactivity observed in light periods in PTZ_7.5_-treated larvae was corrected in a concentration-dependent manner also emerging at Val_2.5_ (Fig. 3a, with Val_5_ also shown in b and c. PTZ_7.5_ = 20.29 cm/min vs. PTZ_7.5_ + Val_2.5_ = 12.85 cm/min, *p* < 0.001; PTZ_7.5_ = 21.13 cm/min vs. PTZ_7.5_ + Val_5_ = 8.42 cm/min, *p* < 0.001; PTZ_7.5_ = 14.10 cm/min vs. PTZ_7.5_ + Val_7_ = 6.85 cm/min, *p* < 0.001). Similarly in dark periods, freezing was corrected in a concentration-dependent manner in co-treatments starting with Val_2.5_ (PTZ_7.5_ = 5.16 cm/min vs. PTZ_7.5_ + Val_2.5_ = 11.34 cm/min, *p* < 0.001; PTZ_7.5_ = 6.48 cm/min vs. PTZ_7.5_ + Val_5_ = 10.66 cm/min, *p* < 0.001; PTZ_7.5_ = 7.18 cm/min vs. PTZ_7.5_ + Val_7_ = 9.09 cm/min, *p* = 0.021). In PTZ_7.5_ + Val_5_ co-treatments in all successive post-transitions PTZ-altered swimming, hyperactivity in light and freezing in dark were both significantly reversed (Fig. 3c and Supplementary Table 7), except for freezing in the last dark period (Fig. 3b, gray box D4), thus clearly highlighting a strong corrective effect in co-treatments with Val_5_. We analyzed further the light-dependent center avoidance at this concentration.

In light periods, lack of center avoidance which we observed in PTZ_7.5_-alone-treated larvae was reversed in PTZ_7.5_ + Val_5_-co-treated larvae in both swim speeds: S2 (Fig. 3f, PTZ_7.5_ = 3.62 cm/min and PTZ_7.5_ + Val_5_ = 1.51 cm/min, *p* < 0.001) and S3 (Fig. 3g, PTZ_7.5_ = 1.96 cm/min vs. PTZ_7.5_ + Val_5_ = 0.57 cm/min, *p* < 0.001). In dark periods, the heightened center avoidance seen in PTZ_7.5_-alone-treated larvae was reversed in PTZ_7.5_ + Val_5_-co-treated larvae in S2 swim speed (Fig. 2f, PTZ_7.5_ = 1.36 vs. PTZ_7.5_ + Val_5_ = 2.60 cm/min, *p* = 0.003). Thus, PTZ-altered light-dependent center avoidance was significantly reversed in at least one swim speed in all successive post-transitions in light and in dark periods (Fig. 3h, S2 and S3 compiled for all successive post-transitions).

## Val_5_ reverses PTZ_7.5_ upregulated transcription of *c-fos*, *npas4a*, and *bdnf* in larval brain

**Fig. 4:**
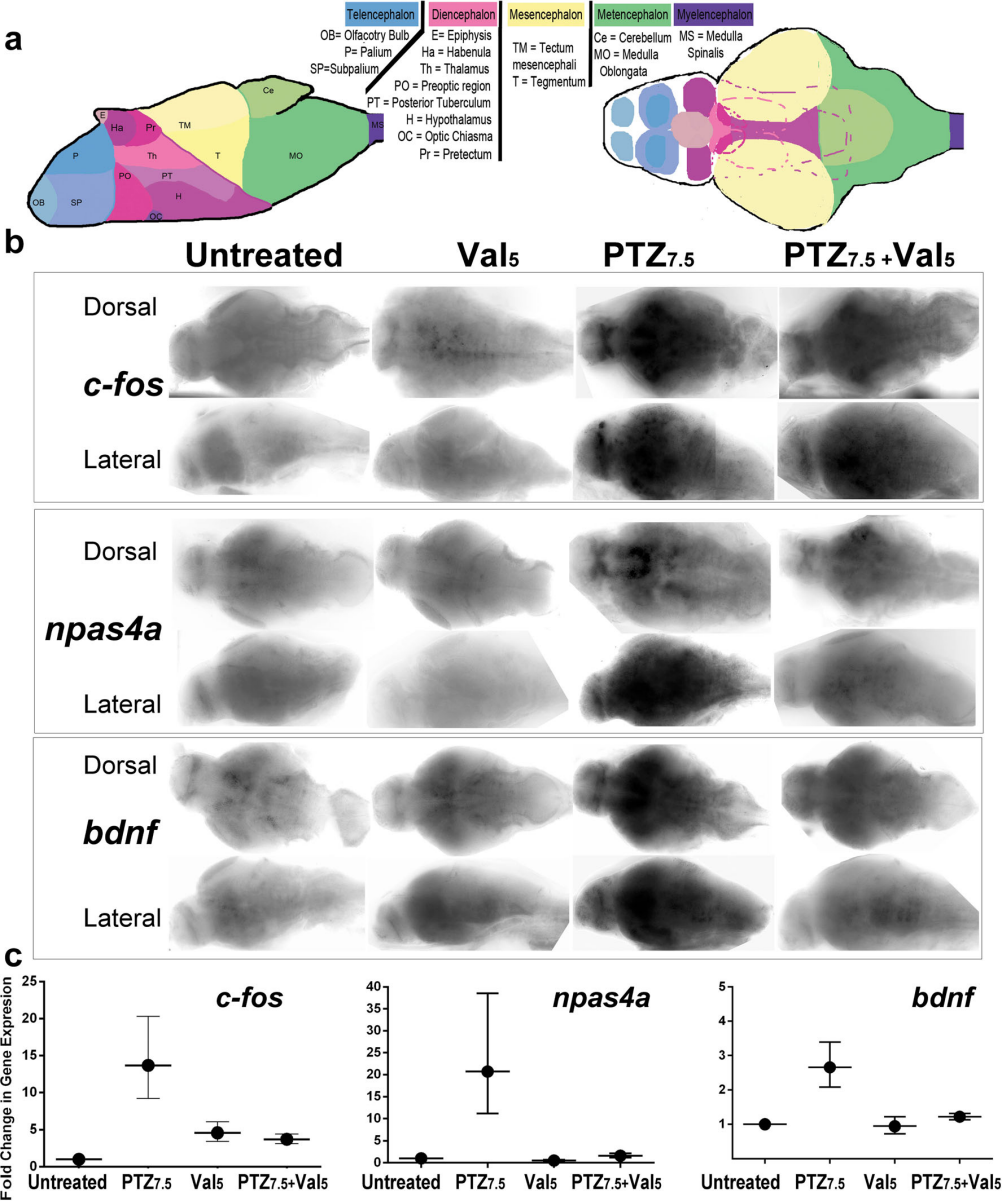
Val_5_ reverses PTZ_7.5_ upregulated transcription of three neural-activity-regulated genes: *c-fos*, *npas4a*, and *bdnf*. **a** Schematics of a lateral view (*left*) and of a dorsal projection (*right)* of a 6-day post-fertilization (dpf) larval brain, which are showing the following: the forebrain or *prosencephalon* (= *telencephalon* in *blue* + *diencephalon* in *pink*), the midbrain (=*mesencephalon* in *yellow*), and the hindbrain or *rhombencephalon* (= *metencephalon* in *green* + *myelencephalon* in *purple*). Identifiable subdomains are described in the *legend* under each brain region. **b** Whole-mount in situ hybridization (WISH) of 6-dpf larvae. Brains have been isolated and mounted in dorsal views (*first*, *third*, and *fifth rows*) and lateral views (*second*, *fourth*, and *sixth rows*). Representative untreated (*first column*), Val_5_-alone-treated (*second column*), PTZ_7.5_-alone-treated (*third column*), and PTZ_7.5_ + Val_5_-co-treated (*fourth column*) larvae are shown. Antisense probes were used against *c-fos* (*two top rows*), *npas4a* (*two middle rows*), and *bdnf* (*two bottom rows*). **c** qPCR measures of transcripts of *c-fos* (*left panel*), *npas4a* (*middle panel*), and *bdnf* (*right panel*). Basal expression was set in untreated (=1) and fold change measured in larvae which had been exposed to PTZ_7.5_-alone, Val_5_-alone, or PTZ_7.5_ + Val_5_. (−) 50 microns in **b**. *Error bars* represent standard deviations (SD) in **c**

To confirm that the reversal of PTZ-altered swimming that we observed in Val treatments was caused by actual changes in brain activity, we checked how the transcriptional regulation of neuronal activity-regulated genes was affected by the different treatments. We assessed transcriptional changes in brains of untreated, PTZ_7.5_-alone-treated, Val_5_-alone-treated, and PTZ_7.5_ + Val_5_-co-treated larvae by WISH (Fig. 4b). We further quantified the changes by qPCRs in whole larvae (Fig. 4c). In particular, we compared gene expression of a small group of selected genes: *c-fos, npas4a, gabra1, gabgr2*, *ppya*, and *bdnf*. These genes had all been previously described as transcriptionally upregulated in PTZ-treated zebrafish larvae (Baxendale et al. 2012).

To analyze the WISH and identify brain regions for each gene expression pattern, we compiled a schematic representation of a larval brain at 6 dpf modified from Wullimann and Knipp (2000), Wullimann and Puelles (1999), and Wullimann et al. (1999, 1996) which we used as a frame of reference (Fig. 4a, lateral view on the left and dorsal projection on the right). We did not see significant changes for *gabra1, gabgr2*, and *ppya* (data not shown), but we did for the three other genes: *c-fos* (Fig. 4b, top panels), *npas4a* (middle panels), and *bdnf* (bottom panels). We found only basal weak expression for all three genes in untreated (first column) and in Val_5_-alone-treated (second column) larvae. As expected, we found a strongly upregulated expression for all three genes in brains of PTZ_7.5_-treated larvae (third column). *The c-fos* gene was strongly expressed in the dorsal and ventral telencephalon (palium and subpalium, respectively), in the diencephalon (preoptic region, posterior tuberculum, and hypothalamus), and in some discrete regions of the hindbrain. The *npas4a* gene was strongly expressed in the ventral telencephalon (subpalium) and ventral diencephalon (posterior tuberculum and hypothalamus), weakly expressed throughout the hindbrain with stronger staining of a subgroup of neurons in the middle/ventral portion of the medulla oblongata. The *c-fos* and *npas4a* expression patterns were only partially overlapping. However, the *bdnf* expression domain seemed mostly comparable to the *npas4a* domain. Strikingly, in PTZ_7.5_ + Val_5_-co-treated animals (fourth column), we saw a drastic reduction in the expression domains of all three genes when compared to the ones found in PTZ_7.5_-alone-treated animals. We found only residual *c-fos* and *npas4a* staining in the ventral telencephalon of a few larvae. Thus, the PTZ-upregulated expression of all three genes was strongly reversed by co-treatments with Val_5_.

To confirm and quantify the transcriptional down-regulation of those three genes, we performed qPCRs (Fig. 4c). We used the basal expression which we measured in untreated animals for each gene to set their expression level at 1. We found that the expression level of *c-fos* (Fig. 4c, left panel) was increased by ∼5-fold inVal_5_-alone-treated animals. This was corroborating the milder hyperactivity seen in the behavioral assay with those treatments. In PTZ_7.5_-alone-treated animals, we found a ∼15-fold increase in gene expression in line with the strong PTZ-altered swimming behavior. Remarkably, in PTZ_7.5_ + Val_5_-co-treated larvae, we found only a ∼5-fold increase in *c-fos* gene expression, similar to what we had found in Val_5_-alone treatments. Next, we analyzed the expression of *npas4a* (middle panel). We found a strong upregulation which was close to ∼20-fold in PTZ_7.5_-alone-treated animals. In PTZ_7.5_ + Val_5_-co-treated and in Val_5_-alone-treated larvae, we measured only basal expression similar to what we had found in untreated larvae. For *bdnf* (right panel), we measured a 3-fold PTZ-induced upregulation, which was not found in co-treatments or Val_5_-alone treatments. Thus, the PTZ-induced upregulation of those two genes was completely abolished in co-treatments, fully corroborating the reversal of PTZ-altered swimming behavior.

## Discussion

In this work, we demonstrate the robustness and sensitivity of a coupled behavioral-molecular approach in zebrafish larvae that we propose as a paradigm to screen at high throughput level complex mixtures of unknown composition for psychoactive efficacy in vivo. As a proxy for measuring psychoactivity, we assessed the reversal of a chemically altered swimming behavior in fish. Similar tests have been proposed to screen for neuro-therapeutics (Ellis and Soanes 2012). First, we showed that when applying PTZ, a commonly used anxiogenic/convulsive compound, at a set concentration (PTZ_7.5_ = 7.5 mM), we could reproducibly alter a strong light-dependent swimming behavior in zebrafish larvae. We focused on three specific parameters during post-transitions of the light conditions which were characteristic of PTZ_7.5_-altered swimming: (1) hyperactivity in the light, (2) freezing in the dark, and (3) altered light-dependent center avoidance. Next, we assessed the reversal of those three parameters by co-treatment with known psychoactive molecule(s). Co-treatments with VPA, a synthetic derivate of valeric acid which is extracted from Val (Patočka 2010), reversed all three parameters in a concentration-dependent manner. More remarkably, co-treatments with crude root extracts of Valerian also reversed all three parameters in a concentration-dependent manner. Thus, we demonstrated the efficacy of the behavioral assay in detecting psychoactive effects in a crude extract prepared from Valerian powdered roots.

The underlying mode of action of reversal of the PTZ-altered swimming remains to be determined. One obvious mode of action could be through the GABAergic system. It is thought that PTZ effects are mainly mediated through inhibition of GABA_A_ receptors (Squires et al. 1984). VPA is known to potentiate GABAergic functions by increasing turnover of gamma-aminobutyric acid (GABA) (for review (Loscher 2002; Loscher and Vetter 1984)). Several lines of evidence show that Valerian extracts bind to the GABA_A_ receptors and might act as an agonist (Cavadas et al. 1995). More recently, it was demonstrated that VPA also has histone deacetylase (HDAC) inhibitory properties (Phiel et al. 2001) linking its neuro-protective effects to modulation of gene expression (Nalivaeva et al. 2009). Future work should explore this possible mode of action for Valerian extracts.

We also found some effects that were treatment-specific. With the higher concentration of VPA (3 mM), VPA_3_-alone-treated larvae were swimming less at all time points measured. In addition, freezing in post-transitions in dark was not significantly reversed in PTZ_7.5_ + VPA_3_-co-treated animals unlike what we had seen with the lower VPA_2_ concentration_._ Both of those effects could be due to sedation, which is a side effect of this drug at high dosage that has been extensively observed in other animal models and in humans (Chateauvieux et al. 2010; Chiu et al. 2013; Nalivaeva et al. 2009). This highlights the sensitivity of our assay and also validates the relevance for human health of potential finding/testing performed in this lower vertebrate.

Noticeably, we did not observe those apparent sedative effects in Val treatments. In fact, we found at all concentrations tested in Val-alone treatments a generalized significant increase in swimming activity at all measured time points, for the exception of treatments with the higher concentration Val_7_ during dark periods. Interestingly, various reliable user reports mention hyperactivity and concentration-dependent variability in effects of Valerian extracts (https://naturalmedicines.therapeuticresearch.com/databases/).

Maybe more surprising was the observation that with the two higher doses of Val, PTZ_7.5_ + Val-co-treated animals behaved in a strikingly similar manner to Val-alone-treated larvae. In other words, co-treated animals were behaving as if PTZ was absent. This was particularly remarkable knowing the strong effects seen in PTZ-alone treatments. This was never observed with VPA. Arguably, we were observing synergic action of the multiple active compounds present in Val extracts. One possible explanation could be that active compounds compete “in vivo” with PTZ for binding to GABA_A_. An alternative explanation could be that active compounds induce conformational modification of the receptors resulting in the inability for PTZ to bind. A strong candidate for exerting one or both of those two effects is valerenic acid (VA) which is a GABA analog and has been implicated as the main active substance in Val extracts in various studies (Becker et al. 2014) (Benke et al. 2009) (Trauner et al. 2008). We tried to assess VA effects with our assay, but even at low dosages VA was highly toxic to larvae. This actually highlights one of the shortcomings of such in vivo assays. As larvae are exposed through their entire body, toxicity because of high dosage and/or off-target effects can be high and secondary effects can mask other results.

In addition to resolving the mechanism underpinning Val potent psychoactive effects, major challenges remain. To cite a few, how representative were the tested samples of Val? Val composition can vary with harvest, season, growth condition, and origin (Patočka 2010). However, that could easily be adjusted by testing different preparations. How does an effective dosage in zebrafish larvae translate to an adequate posology in humans? This is a real challenge as the mode of administration can drastically change the composition of the final product through digestion or filtering through the blood-brain barrier (BBB). In zebrafish, a BBB is present as early as 3 dpf and at least partially functional (Eliceiri et al. 2011). Interestingly, Val extracts have been shown to contain high quantities of GABA, but it is generally admitted that those would not cross the BBB. This could be tested by labeling GABA molecules and observing their distribution after exposure in zebrafish larvae.

In parallel to behavioral measures, we measured transcriptional changes in a small number of known synaptic activity-induced genes, known to be regulated by PTZ treatments (Baxendale et al. 2012). Our molecular data strongly corroborated our behavioral data but also provided the first molecular evidence of the psychoactive potency of a plant crude extract. *C-fos* has become the gold standard for detecting synaptic activity (Zhang et al. 2002). We showed that *c-fos* upregulation was 3-fold lower in co-treatments than in PTZ-alone treatments, which correlated nicely with the reversal of PTZ-altered swimming activity. Furthermore, levels of gene expression of *c-fos* were similar in Val_5_-alone-treated and in PTZ_7.5_ + Val_5_-co-treated animals, which again corroborated the striking similarity in behavior that we had observed in those two treatments. *Npas4a*, a more recently discovered transcription factor, is involved in regulating excitatory-inhibitory balance within neural circuits (Bloodgood et al. 2013; Lin et al. 2008; Spiegel et al. 2014). Its importance in neurological disorders including bipolar disorders was clearly established but not fully elucidated (Coutellier et al. 2012; Jaehne et al. 2015). One putative downstream target of *c-fos* and *npas4a* is the neurotrophic factor *bdnf* (Greenberg et al. 2009; Lin et al. 2008). Neuronal activity-dependent upregulation of *bdnf* is well known (Zhang et al. 2002), and both GABA_A_ receptors can be modulated by *bdnf* (Marty et al. 2000). We found that both *npas4a* and *bdnf* were transcriptionally regulated by Val treatments. Three other genes (*ppya*, *gabra1*, and *gbrg2*) which are widely expressed in zebrafish larval brain as early as 2 dpf (Baxendale et al. 2012) did not show a significant regulation with either methods, WISH or qPCRs, maybe because time of exposure to PTZ was too short. Both *c-fos* and *npas4a* are immediate early response (IER) genes which are activated within minutes and without needing protein neo-synthesis (Flavell and Greenberg 2008). By comparison, *bdnf* will only be induced after at least 1 h of exposure (Flavell and Greenberg 2008) (Marty et al. 2000). Consistent with this, we found that *bdnf* was the least activated gene of the three.

It will be of importance to effectively decorticate gene cascades involved in the corrective action of any psychoactive drug isolated with a screen such as the one we describe here. However, for now, a major limiting factor is the fact that larval zebrafish brain is still very poorly documented. Precise localization of gene expression domains is indispensable to understand which neural circuitries are involved. This problem should be resolving as a growing number of laboratories are working with this animal model.

Nevertheless, our findings suggest high conservation in neural circuitries governing anxiety and mood-stabilizing mechanisms from fish to humans. This validated our low-cost, potent screening platform for neuroactive compounds which could be detected even in a crude plant preparation. This screening tool will advance discovery of new psychoactive molecules because it worked with minimal preparation/standardization of plant extracts and easy drug application. Ultimately, this work can be extended to test any ethnobotanical plant of interest. Once crude extracts show psychoactivity, successive purification steps or micro-fractionation to isolate bioactive component(s) can be done (Challal et al. 2014); (Bohni et al. 2013). In vivo competition-binding assays with agonist/antagonist of specific receptors could be another continuation. In addition to dissect drug targets, this assay can be performed in mutant larvae which are defective for a given brain receptor for example. The combination of behavioral-molecular assays with the genetic power of this model will undoubtedly advance drug discovery.

## Electronic supplementary material

Below is the link to the electronic supplementary material.

Table 1 (DOCX 22 kb)

Table 2 (DOCX 43 kb)

Table 3 (DOCX 25 kb)

Table 4 (DOCX 25 kb)

Table 5 (DOCX 25 kb)

Table 6 (DOCX 43 kb)

Table 7 (DOCX 24 kb)

Table 8 (DOCX 24 kb)

Table 9 (DOCX 24 kb)
