# Supplementary material for: Reversal of pentylenetetrazole-altered swimming and neural activity-regulated gene expression in zebrafish larvae by valproic acid and valerian extract
Source: Psychopharmacology (Berl). 2016 May 11;233:2533–47. doi: 10.1007/s00213-016-4304-z (PMC4908174; doi:10.1007/s00213-016-4304-z)
Supplement: Supplementary file 1 — (DOCX 22 kb) [file 213_2016_4304_MOESM1_ESM.docx]

**Supplementary Tables**

**Table 1** STATA analyses of distances traveled by untreated (Unt) vs. PTZ_7.5_-treated larvae. (Fig.1)

| **Figure #, whole well (WW)/inner space (IS)** | **Swim speed (S2/S3);**  **Light pattern (Light (L)/Dark(D);**  **time segment (entire period/Post-transition/Non-transition)** | **Treatment** | **Mean** | **[SEM]** | **p value** |
| --- | --- | --- | --- | --- | --- |
| 1a  WW | S2 in D (all 4 entire cycles)  D1 (11-20) + D2 (31-40) + D3 (51-60) + D4 (71-80) | Unt  PTZ | 11.25  7.96 | 0.12  0.16 | <0.001 |
| 1a  WW | S2 in L (all 4 entire cycles)  L1 (1-10) + L2 (21-30) + L3 (41-50) + L4 (61-70) | Unt  PTZ | 4.02  15.52 | 0.08  0.14 | <0.001 |
|  |  |  |  |  |  |
| 1c  WW | S2 in D all first minutes  = Post-transition  D1 (+11) + D2 (+31) + D3 (+51) + D4 (+61) | Unt  PTZ | 12.47  6.19 | 0.37  0.36 | <0.001 |
| 1c  WW | S2 in L all first minutes  = Post-transition  L1 (+1) + L2 (+21) + L3 (+41) + L4 (+51) | Unt  PTZ | 4.54  19.17 | 0.24  0.46 | <0.001 |
|  |  |  |  |  |  |
| 1e  WW | S2 in D all non-first minutes  = Non-transition  D1 (12-20) + D2 (32-40) + D3 (52-60) + D4 (72-80) | Unt  PTZ | 11.11  8.16 | 0.12  0.18 | <0.001 |
| 1e  WW | S2 in L all non-first minutes  = Non-transition  L1 (2-10) + L2 (22-30) + L3 (42-50) + L4 (62-70) | Unt  PTZ | 3.96  15.12 | 0.09  0.15 | <0.001 |
|  |  |  |  |  |  |
| 1f  IS | S2 in D all first minutes  = Post-transition  D1 (+11) + D2 (+31) + D3 (+51) + D4 (+61) | Unt  PTZ | 5.18  0.46 | 0.22  0.08 | <0.001 |
| 1f  IS | S2 in L all first minutes  = Post-transition  L1 (+1) + L2 (+21) + L3 (+41) + L4 (+51) | Unt  PTZ | 1.08  0.96 | 0.13  0.09 | 0.584 |
|  |  |  |  |  |  |
| 1g  IS | S3 in D all first minutes  = Post-transition  D1 (+11) + D2 (+31) + D3 (+51) + D4 (+61) | Unt  PTZ | 0.57  0.32 | 0.04  0.03 | <0.001 |
| 1g  IS | S3 in L all first minutes  = Post-transition  L1 (+1) + L2 (+21) + L3 (+41) + L4 (+51) | Unt  PTZ | 0.02  1.03 | 0.01  0.07 | <0.001 |
