# Supplementary material for: Reversal of pentylenetetrazole-altered swimming and neural activity-regulated gene expression in zebrafish larvae by valproic acid and valerian extract
Source: Psychopharmacology (Berl). 2016 May 11;233:2533–47. doi: 10.1007/s00213-016-4304-z (PMC4908174; doi:10.1007/s00213-016-4304-z)
Supplement: Supplementary file 2 — (DOCX 43 kb) [file 213_2016_4304_MOESM2_ESM.docx]

**Table 2** STATA analyses of distances traveled by untreated (Unt) vs. (PTZ_7.5_, VPA, VPA+ PTZ_7.5_-treated) larvae (Fig.2)

| **[VPA]** | **Figure #, whole well (WW)/inner space (IS)** | **Swim speed (S2/S3), Light Pattern (light(L)/Dark(D) and time segment(entire period/post-transition/non-transition)** | **Treatment** | **Mean** | **SEM** | **p value**  **Ref Unt** | **p value**  **Ref PTZ** | **p value**  **Ref VPA** |
| --- | --- | --- | --- | --- | --- | --- | --- | --- |
| **VPA_05_** | 2a  WW | S2 in D (all 4 entire cycles)  D1 (11-20) + D2 (31-40) + D3 (51-60) + D4 (71-80) | Unt  PTZ_7.5_  VPA_0.5_  VPA_0.5_+PTZ_7.5_ | 10.31  7.54  10.87  7.04 | 0.15  0.22  0.12  0.23 | 0.029  0.280  0.002 | 0.117  0.750 | <0.001 |
| **VPA_0.5_** | 2a  WW | S2 in L (all 4 entire cycles)  L1 (1-10) + L2 (21-30) + L3 (41-50) + L4 (61-70) | Unt  PTZ_7.5_  VPA_0.5_  VPA_0.5_+PTZ_7.5_ | 2.94  15.13  2.06  12.53 | 0.10  0.21  0.07  0.20 | <0.001  0.807  <0.001 | <0.001  0.002 | <0.001 |
|  |  |  |  |  |  |  |  |  |
| **VPA_0.5_** | 2a  WW | S2 in D all first minutes  = Post-transition  D1 (+11) + D2 (+31) + D3 (+51) + D4 (+71) | Unt  PTZ_7.5_  VPA_0.5_  VPA_0.5_+PTZ_7.5_ | 12.28  6.29  11.86  7.03 | 0.45  0.49  0.32  0.45 | <0.001  0.465  <0.001 | <0.001  0.439 | <0.001 |
| **VPA_0.5_** | 2a  WW | S2 in L all first minutes  = Post-transition  L1 (+1) + L2 (+21) + L3 (+41) + L4 (+61) | Unt (U)  PTZ_7.5_  VPA_0.5_  VPA_0.5_+PTZ_7.5_ | 4.18  21.46  3.88  14.82 | 0.38  0.65  0.24  0.65 | < 0.001  0.689  <0.001 | <0.001  <0.001 | <0.001 |
|  |  |  |  |  |  |  |  |  |
| **VPA_0.5_** | 2a  WW | S2 in D all non-first minutes  = Non-transition  D1 (12-20) + D2 (32-40) + D3 (52-60) + D4 (72-80) | Unt  PTZ_7.5_  VPA_0.5_  VPA_0.5_+PTZ_7.5_ | 10.10  7.68  10.76  7.04 | 0.16  0.23  0.13  0.25 | 0.083  0.325  <0.011 | 0.260  0.696 | <0.001 |
| **VPA_0.5_** | 2a  WW | S2 in L all non-first minutes  = Non-transition  L1 (2-10) + L2 (22-30) + L3 (42-50) + L4 (62-70) | Unt  PTZ_7.5_  VPA_0.5_  VPA_0.5_+PTZ_7.5_ | 2.80  14.43  1.86  12.27 | 0.11  0.21  0.07  0.21 | <0.001  0.730  <0.001 | <0.001  0.016 | <0.001  <0.001 |
|  |  |  |  |  |  |  |  |  |
| **VPA_1_** | 2a  WW | S2 in D (all 4 entire cycles)  D1 (11-20) + D2 (31-40) + D3 (51-60) + D4 (71-80) | Unt  PTZ_7.5_  VPA_1_  VPA_1_+PTZ_7.5_ | 11.83  7.78  10.81  5.83 | 0.13  0.22  0.12  0.19 | <0.001  0.584  <0.001 | <0.001  0.008 | <0.001 |
| **VPA_1_** | 2a  WW | S2 in L (all 4 entire cycles)  L1 (1-10) + L2 (21-30) + L3 (41-50) + L4 (61-70) | Unt  PTZ_7.5_  VPA_1_  VPA_1_+PTZ_7.5_ | 3.62  15.12  2.21  10.39 | 0.11  0.21  0.08  0.20 | <0.001  0.607  <0.001 | <0.001  <0.001 | <0.001 |
|  |  |  |  |  |  |  |  |  |
| **VPA_1_** | 2a  WW | S2 in D all first minutes  = Post-transition  D1 (+11) + D2 (+31) + D3 (+51) + D4 (+71) | Unt  PTZ_7.5_  VPA_1_  VPA_1_+PTZ_7.5_ | 14.51  6.38  11.22  7.30 | 0.36  0.51  0.30  0.52 | < 0.001  < 0.001  < 0.001 | < 0.001  0.262 | <0.001 |
| **VPA_1_** | 2a  WW | S2 in L all first minutes  = Post-transition  L1 (+1) + L2 (+21) + L3 (+41) + L4 (+61) | Unt  PTZ_7.5_  VPA_1_  VPA_1_+PTZ_7.5_ | 4.09  21.90  4.36  10.95 | 0.31  0.64  0.33  0.54 | <0.001  0.553  <0.001 | <0.001  <0.001 | <0.001 |
|  |  |  |  |  |  |  |  |  |
| **VPA_1_** | 2a  WW | S2 in D all non-first minutes  = Non-transition  D1 (12-20) + D2 (32-40) + D3 (52-60) + D4 (72-80) | Unt  PTZ_7.5_  VPA_1_  VPA_1_+PTZ_7.5_ | 11.53  7.93  10.77  5.67 | 0.13  0.24  0.13  0.20 | 0.007  0.840  <0.001 | <0.001  0.003 | <0.001 |
| **VPA_1_** | 2a  WW | S2 in L all non-first minutes  = Non-transition  L1 (2-10) + L2 (22-30) + L3 (42-50) + L4 (62-70) | Unt  PTZ_7.5_  VPA_1_  VPA_1_+PTZ_7.5_ | 3.57  14.37  1.98  10.33 | 0.12  0.22  0.08  0.22 | <0.001  0.500  <0.001 | <0.001  <0.001 | <0.001 |
|  |  |  |  |  |  |  |  |  |
| **VPA_2_** | 2a  WW | S2 in D (all 4 entire cycles)  D1 (11-20) + D2 (31-40) + D3 (51-60) + D4 (71-80) | Unt  PTZ_7.5_  VPA_2_  VPA_2_+PTZ_7._ | 10.75  8.96  8.15  4.34 | 0.16  0.21  0.12  0.12 | 0.098  <0.001  <0.001 | 0.067  <0.001 | <0.001 |
| **VPA_2_** | 2a  WWl | S2 in L (in all 4 entire cycles)  L1 (1-10) + L2 (21-30) + L3 (41-50) + L4 (61-70) | Unt  PTZ_7.5_  VPA_2_  VPA_2_+PTZ_7.5_ | 3.36  14.65  2.44  5.44 | 0.11  0.20  0.07  0.13 | <0.001  0.156  0.004 | <0.001  <0.001 | <0.001 |
|  |  |  |  |  |  |  |  |  |
| **VPA_2_** | 2c  Whole well | S2 in D all first minutes  = Post-transition  D1 (+11) + D2 (+31) + D3 (+51) + D4 (+71) | Unt  PTZ_7.5_  VPA_2_  VPA_2_+PTZ_7.5_ | 14.28  5.61  9.86  7.55 | 0.43  0.48  0.34  0.43 | <0.001  <0.001  <0.001 | <0.001  <0.001 | <0.001 |
| **VPA_2_** | 2c  WW | S2 in L all first minutes  = Post-transition  L1 (+1) + L2 (+21) + L3 (+41) + L4 (+61) | Unt  PTZ_7.5_  VPA_2_  VPA_2_+PTZ_7.5_ | 4.59  19.95  4.42  6.96 | 0.45  0.60  0.27  0.36 | <0.001  0.672  0.026 | <0.001  <0.001 | <0.001 |
|  |  |  |  |  |  |  |  |  |
| **VPA_2_** | 2e  WW | S2 in D all non-first minutes  = Non-transition  D1 (12-20) + D2 (32-40) + D3 (52-60) + D4 (72-80) | Unt  PTZ_7.5_  VPA_2_  VPA_2_+PTZ_7._ | 10.36  9.33  7.96  3.98 | 0.17  0.22  0.12  0.12 | 0.364  <0.001  <0.001 | 0.013  <0.001 | <0.001 |
| **VPA_2_** | 2e  WW | S2 in L all non-first minutes  = Non-transition  L1 (2-10) + L2 (22-30) + L3 (42-50) + L4 (62-70) | Unt  PTZ_7.5_  VPA_2_  VPA_2_+PTZ_7.5_ | 3.22  14.07  2.22  5.37 | 0.11  0.20  0.07  0.14 | <0.001  0.176  0.005 | <0.001  <0.001 | <0.001 |
|  |  |  |  |  |  |  |  |  |
| **VPA_3_** | 2a  WW | S2 in Dark (all 4 entire cycles)  D1 (11-20) + D2 (31-40) + D3 (51-60) + D4 (71-80) | Unt  PTZ_7.5_  VPA_3_  VPA_3_+PTZ_7.5_ | 9.02  8.96  5.63  5.43 | 0.17  0.19  0.11  0.14 | 0.960  <0.001  <0.001 | 0.002  <0.001 | <0.001 |
| **VPA_3_** | 2a  WW | S2 in L (all 4 entire cycles)  L1 (1-10) + L2 (21-30) + L3 (41-50) + L4 (61-70) | Unt  PTZ_7.5_  VPA_3_  VPA_3_+PTZ_7.5_ | 2.78  13.61  1.22  6.07 | 0.10  0.19  0.05  0.14 | <0.001  0.166  <0.001 | <0.001  <0.001 | <0.001 |
|  |  |  |  |  |  |  |  |  |
| **VPA_3_** | 2a  WW | S2 in D all first minutes  = Post-transition  D1 (+11) + D2 (+31) + D3 (+51) + D4 (+71) | Unt  PTZ_7.5_  VPA_3_  VPA_3_+ PTZ_7.5_ | 12.87  5.27  5.68  6.65 | 0.53  0.43  0.31  0.44 | <0.001  <0.001  <0.001 | 0.478  0.549 | 0.893 |
| **VPA_3_** | 2a  WW | S2 in L all first minutes  = Post-Transition  L1 (+1) + L2 (+21) + L3 (+41) + L4 (+61) | Unt  PTZ_7.5_  VPA_3_  VPA_3_+PTZ_7.5_ | 4.30  17.21  2.94  6.19 | 0.45  0.64  0.24  0.41 | <0.001  0.166  0.062 | <0.001  <0.001 | <0.001 |
|  |  |  |  |  |  |  |  |  |
| **VPA_3_** | 2a  WW | S2 in D all non-first minutes  = Non-transition  D1 (12-20) + D2 (32-40) + D3 (52-60) + D4 (72-80) | Unt  PTZ_7.5_  VPA_3_  VPA_3_+PTZ_7.5_ | 8.59  9.37  5.63  5.30 | 0.17  0.21  0.11  0.14 | 0.468  0.007  <0.001 | <0.001  <0.001 | <0.001 |
| **VPA_3_** | 2a  WW | S2 in L all non-first minutes  = Non-transition  L1 (2-10) + L2 (22-30) + L3 (42-50) + L4 (62-70) | Unt  PTZ_7.5_  VPA_3_  VPA_3_+PTZ_7.5_ | 2.61  13.22  1.03  6.05 | 0.10  0.20  0.05  0.15 | <0.001  0.156  <0.001 | <0.001  <0.001 | <0.001 |
|  |  |  |  |  |  |  |  |  |
| **VPA_2_** | 2f  IS | S2 in D all first minutes  = Post-transition  D1 (+11) + D2 (+31) + D3 (+51) + D4 (+61) | Unt  PTZ_7.5_  VPA_2_  VPA_2_+PTZ_7.5_ | 5.03  0.48  2.65  1.27 | 0.25  0.09  0.15  0.14 | <0.001  <0.001  <0.001 | <0.001  0.008 | <0.001 |
| **VPA_2_** | 2f  IS | S2 in L all first minutes  = Post-transition  L1 (+1) + L2 (+21) + L3 (+41) + L4 (+51) | Unt  PTZ_7.5_  VPA_2_  VPA_2_+PTZ_7._ | 0.61  1.12  0.40  0.39 | 0.15  0.13  0.08  0.06 | <0.001  0.137  0.124 | 0.001  <0.001 | 0.954 |
|  |  |  |  |  |  |  |  |  |
| **VPA_2_** | 2g  IS | S3 in D all first minutes  = Post-transition  D1 (+11) + D2 (+31) + D3 (+51) + D4 (+61) | Unt  PTZ_7.5_  VPA_2_  VPA_2_+PTZ_7.5_ | 0.32  0.35  0.13  0.47 | 0.05  0.05  0.02  0.06 | 0.800  0.028  0.111 | 0.013  0.193 | <0.001 |
| **VPA_2_** | 2g  IS | S3 in L all first minutes  = Post-transition  L1 (+1) + L2 (+21) + L3 (+41) + L4 (+51) | Unt  PTZ_7.5_  VPA_2_  VPA_2_+PTZ_7.5_ | 0.02  1.44  0.04  0.31 | 0.01  0.13  0.02  0.04 | 0.001  0.924  0.006 | <0.001  <0.001 | <0.001 |
