# Supplementary material for: Reversal of pentylenetetrazole-altered swimming and neural activity-regulated gene expression in zebrafish larvae by valproic acid and valerian extract
Source: Psychopharmacology (Berl). 2016 May 11;233:2533–47. doi: 10.1007/s00213-016-4304-z (PMC4908174; doi:10.1007/s00213-016-4304-z)
Supplement: Supplementary file 3 — (DOCX 25 kb) [file 213_2016_4304_MOESM3_ESM.docx]

**Table 3** STATA analyses of total distances traveled in swim speed S2 by untreated (Unt) vs. (PTZ_7.5_, VPA_2_, VPA_2_+ PTZ_7.5_-treated) larvae during all successive transitions (Fig.2b)

**Note**: We used a modified Brown and Forysthe test giving results in the format of a 95% Confidence Intervals (CI). When 0 (zero) is not included in the CI the result is considered significant

| **Fig.2b**  **all transitions**  **whole plate (WW)**  **in S2** | **Treatment** | **Mean** | **SEM** | **95% CI**  **Ref Unt** | **95% CI**  **Ref PTZ_7.5_** | **95% CI**  **Ref VPA_2_** |
| --- | --- | --- | --- | --- | --- | --- |
| L1 (min1) | Unt  PTZ_7.5_  VPA_2_  VPA_2_+PTZ_7.5_ | 3.43  15.38  5.76  6.07 | 0.47  1.06  0.49  0.60 | -15.40 – -7.52  -4.15 – 0.34  -4.59 – 0.26 | 5.62 – 13.60  5.26 – 13.38 | -2.69 – 2.17 |
| D1 (min11) | Unt  PTZ_7.5_  VPA_2_  VPA_2_+PTZ_7.5_ | 13.80  6.28  9.00  7.51 | 1.04  1.01  0.57  0.78 | 2.45 – 11.40  1.30 – 8.14  2.72 – 10.30 | -6.13 – 1.66  -4.65 – 3.80 | -1.21 – 4.82 |
| L2 (min21) | Unt  PTZ_7.5_  VPA_2_  VPA_2_+PTZ_7.5_ | 3.99  22.17  4.91  5.22 | 0.67  1.11  0.62  0.66 | -22.20 – -14.20  -3.34 – 2.11  -4.17 – 1.78 | 13.78 – 21.40  13.02 – 21.00 | -3.24 – 2.08 |
| D2 (min31) | Unt  PTZ_7.5_  VPA_2_  VPA_2_+PTZ_7.5_ | 14.72  4.45  10.19  7.21 | 0.73  0.66  0.67  0.74 | 7.02 – 13.40  1.72 – 7.76  4.75 – 11.10 | -8.53 – -2.38  -5.50 – 0.948 | 0.11 – 6.25 |
| L3 (min41) | Unt  PTZ_7.5_  VPA_2_  VPA_2_+PTZ_7.5_ | 5.66  21.47  3.40  5.61 | 1.20  1.02  0.45  5.61 | -20.40 – -10.8  -1.79 – 6.16  -4.17 – 4.18 | 14.3 – 21.20  11.90 – 19.31 | -4.57 – 0.21 |
| D3 (min51) | Unt  PTZ_7.5_  VPA_2_  VPA_2_+PTZ_7.5_ | 14.59  5.57  10.65  8.17 | 0.83  0.72  0.56  0.75 | 5.60 – 12.30  1.15 – 7.01  3.20 – 9.96 | -7.77 – -1.91  -5.73 – 1.04 | -0.50– 5.45 |
| L4 (min61) | Unt  PTZ_7.5_  VPA_2_  VPA_2_+PTZ_7.5_ | 3.97  20.56  3.79  7.41 | 0.73  0.76  0.40  0.71 | -19.70 – -12.6  -1.80 – 3.82  -6.04 – 0.67 | 14.40 – 19.98  10.12 – 16.83 | -6.27 – -1.12 |
| D4 (min71) | Unt  PTZ_7.5_  VPA_2_  VPA_2_+PTZ_7.5_ | 13.64  5.32  9.93  8.01 | 0.77  0.87  0.67  0.83 | 4.88 – 12.30  0.982 – 7.28  2.310 – 9.44 | -8.02 – -0.86  -6.64 – 1.25 | -1.70 – 5.19 |
