# Supplementary material for: Reversal of pentylenetetrazole-altered swimming and neural activity-regulated gene expression in zebrafish larvae by valproic acid and valerian extract
Source: Psychopharmacology (Berl). 2016 May 11;233:2533–47. doi: 10.1007/s00213-016-4304-z (PMC4908174; doi:10.1007/s00213-016-4304-z)
Supplement: Supplementary file 4 — (DOCX 25 kb) [file 213_2016_4304_MOESM4_ESM.docx]

**Table 4** STATA analyses of inner distances traveled in swim speed S2 by untreated (Unt) versus (PTZ_7.5_, VPA_2_, VPA_2_+ PTZ_7.5_-treated) larvae during all successive transitions (Fig.2h)

**Note**: We used a modified Brown and Forysthe test giving results in the format of a 95% Confidence Intervals (CI). When 0 (zero) is not included in the CI the result is considered significant

| **Fig.2h all transitions**  **inner space**  **(IS)**  **in S2** | **Treatment** | **Mean** | **SEM** | **95% CI**  **Ref Unt** | **95% CI**  **Ref PTZ** | **95% CI**  **Ref VPA_2_** |
| --- | --- | --- | --- | --- | --- | --- |
| L1 (min1) | Unt  PTZ_7.5_  VPA_2_  VPA_2_+PTZ_7.5_ | 0.96  0.77  0.75  0.55 | 0.33  0.19  0.17  0.16 | -0.923 – 1.292  -0.877 – 1.293  -0.666 – 1.474 | -0.727 – 0.775  -0.507 – 0.947 | -0.491 – 0.883 |
| D1 (min11) | Unt  PTZ_7.5_  VPA_2_  VPA_2_+PTZ_7.5_ | 4.65  1.15  2.18  0.79 | 0.53  0.27  0. 26  0.20 | 1.763 – 5.237  0.744 – 4.206  2.196 – 5.534 | -2.116 – 0.066  -0.616 – 1.345 | 0.424 – 2.355 |
| L2 (min21) | Unt  PTZ_7.5_  VPA_2_  VPA_2_+PTZ_7.5_ | 0.81  0.96  0.41  0.23 | 0.46  0.20  0.20  0.06 | -1.622 – 1.315  -1.065 – 1.875  -0.798 – 1.954 | -0.268 – 1.385  0.104 – 1.359 | -0.451 – 0.796 |
| D2 (min31) | Unt  PTZ_7.5_  VPA_2_  VPA_2_+PTZ_7.5_ | 5.15  0.24  2.35  1.39 | 0.50  0.06  0.29  0.33 | 3.414 – 6.407  1.112 – 4.471  2.013 – 5.504 | -2.977 – -1.258  -2.146 – -0.156 | -0.301 – 2.233 |
| L3 (min41) | Unt  PTZ_7.5_  VPA_2_  VPA_2_+PTZ_7.5_ | 0.23  1.33  0.10  0.36 | 0.11  0.31  0.04  0.10 | -2.064 – -0.136  -0.214 – 0.477  -0.560 – 0.305 | 0.305 – 2.157  0.014 – 1.932 | -0.580 – 0.064 |
| D3 (min51) | Unt  PTZ_7.5_  VPA_2_  VPA_2_+PTZ_7.5_ | 5.28  0.47  2.97  1.61 | 0.49  0.15  0.28  0.31 | 3.297 – 6.321  0.670 – 3.953  1.984 – 5.351 | -3.418 – -1.578  -2.142 – -0.141 | 0.157 – 2.555 |
| L4 (min61) | Unt  PTZ_7.5_  VPA_2_  VPA_2_+PTZ_7.5_ | 0.41  1.38  0.31  0.40 | 0.19  0.28  0.11  0.11 | -1.983 – 0.402  -0.566 – 0.760  -0.641 – 0.673 | 0.160 – 1.977  0.083 – 1.892 | -0.537 – 0.374 |
| D4 (min71) | Unt  PTZ_7.5_  VPA_2_  VPA_2_+PTZ_7.5_ | 5.02  0.05  3.09  1.28 | 0.44  0.02  0.37  0.24 | 3.659– 6.279  0.275 – 3.589  2.281 – 5.196 | -4.115 – -1.958  -1.927 – -0.533 | 0.544 – 3.068 |
