# Supplementary material for: Reversal of pentylenetetrazole-altered swimming and neural activity-regulated gene expression in zebrafish larvae by valproic acid and valerian extract
Source: Psychopharmacology (Berl). 2016 May 11;233:2533–47. doi: 10.1007/s00213-016-4304-z (PMC4908174; doi:10.1007/s00213-016-4304-z)
Supplement: Supplementary file 5 — (DOCX 25 kb) [file 213_2016_4304_MOESM5_ESM.docx]

**Table 5** STATA analyses of inner distances traveled in swim speed S3 by untreated (Unt) vs. (PTZ_7.5_, VPA_2_, VPA_2_+ PTZ_7.5_-treated) larvae during all successive transitions (Fig.2h)

**Note**: We used a modified Brown and Forysthe test giving results in the format of a 95% Confidence Intervals (CI). When 0 (zero) is not included in the CI the result is considered significant

| **Fig.2h all transitions**  **whole plate (WW)**  **in S2** | **Treatment** | **Mean** | **SEM** | **95% CI**  **Ref Unt** | **95% CI**  **Ref PTZ** | **95% CI**  **Ref VPA_2_** |
| --- | --- | --- | --- | --- | --- | --- |
| L1 (min1) | Unt  PTZ_7.5_  VPA_2_  VPA_2_+PTZ_7.5_ | 0.02  0.87  0.04  0.36 | 0.01  0.22  0.02  0.08 | -1.53 – 0.17  - 0.12 – 0.07  -0.59 – 0.08 | 0.15 – 1.51  -0.20 – 1.23 | -0.58 – -0.05 |
| D1 (min11) | Unt  PTZ_7.5_  VPA_2_  VPA_2_+PTZ_7.5_ | 0.37  0.65  0.11  0.52 | 0.10  0.14  0.04  0.12 | -0.80 – -0.23  -0.08 – 0.56  -0.62 – 0.31 | 0.10 – 0.98  -0.41 – 0.66 | -0.78 – -0.46 |
| L2 (min21) | Unt  PTZ_7.5_  VPA_2_  VPA_2_+PTZ_7.5_ | 0.00  1.51  0.05  0.36 | 0.00  0.22  0.04  0.08 | -2.18 – -0.85  -0.18 – 0.08  -0.63 – -0.10 | 0.79 – 2.14  0.45 – 1.86 | -0.59– -0.02 |
| D2 (min31) | Unt  PTZ_7.5_  VPA_2_  VPA_2_+PTZ_7.5_ | 0.30  0.30  0.10  0.43 | 0.10  0.12  0.03  0.11 | -0.45 – 0.47  -0.11 – 0.53  -0.57 – 0.31 | -0.17 – 0.57  -0.62 – 0.34 | -0.68 – 0.12 |
| L3 (min41) | Unt  PTZ_7.5_  VPA_2_  VPA_2_+PTZ_7.5_ | 0.01  1.52  0.04  0.29 | 0.01  0.20  0.03  0.08 | -2.12 – -0.89  -0.15 – 0.10  -0.54 – -0.01 | 0.85 – 2.10  0.57 – 1.89 | -0.53 – 0.03 |
| D3 (min51) | Unt  PTZ_7.5_  VPA_2_  VPA_2_+PTZ_7.5_ | 0.24  0.26  0.16  0.44 | 0.07  0.06  0.05  0.12 | -0.31 – 0.27  -0.19 – 0.34  -0.61 – 0.21 | -0.14 – 0.34  -0.58 – 0.22 | -0.66 – 0.10 |
| L4 (min61) | Unt  PTZ_7.5_  VPA_2_  VPA_2_+PTZ_7.5_ | 0.02  1.85  0.02  0.22 | 0.01  0.30  0.01  0.07 | -2.74 – -0.92  -0.07 – 0.07  -0.42 – 0.03 | 0.92 – 2.73  0.70 – 2.56 | -0.42 – 0.03 |
| D4 (min71) | Unt  PTZ_7.5_  VPA_2_  VPA_2_ +PTZ_7.5_ | 0.35  0.15  0.14  0.45 | 0. 10  0.05  0.05  0.09 | -0.14– 0.52  -0.12 – 0.52  -0.51 –0.30 | -0.20 – 0.23  -0.62 – 0.02 | -0.63 – 0.01 |
