# Supplementary material for: Reversal of pentylenetetrazole-altered swimming and neural activity-regulated gene expression in zebrafish larvae by valproic acid and valerian extract
Source: Psychopharmacology (Berl). 2016 May 11;233:2533–47. doi: 10.1007/s00213-016-4304-z (PMC4908174; doi:10.1007/s00213-016-4304-z)
Supplement: Supplementary file 6 — (DOCX 43 kb) [file 213_2016_4304_MOESM6_ESM.docx]

**Table 6** STATA analyses of distances traveled by untreated (Unt) vs. (PTZ_7.5_, Val, Val+ PTZ_7.5_-treated) larvae (Fig.3)

| **[Val]** | **Figure #, whole well (WW) /inner space (IS)** | **Swim Speed (S2/S3), Light Pattern (light(L)/Dark(D) and time segment(entire period/Post-transition/Non-transition)** | **Treatment** | **Mean** | **SEM** | **p value**  **Ref Unt** | **p value**  **Ref PTZ** | **p value**  **Ref Val** |
| --- | --- | --- | --- | --- | --- | --- | --- | --- |
| Val_1_ | 3a  WW | S2 in D (in all 4 entire 4 cycles)  D1 (11-20) + D2 (31-40) + D3 (51-60) + D4 (71-80) | Unt  PTZ_7.5_  Val_1_  Val_1_+PTZ_7.5_ | 10.24  8.90  15.09  9.04 | 0.17  0.23  0.13  0.14 | 0.180  <0.001  0.167 | <0.001  0.868 | <0.001 |
| Val_1_ | 3a  WW | S2 in L (in all 4 entire cycles)  L1 (1-10) + L2 (21-30) + L3 (41-50) + L4 (61-70) | Unt  PTZ_7.5_  Val_1_  Val_1_ + PTZ_7.5_ | 2.88  16.14  4.59  15.09 | 0.12  0.19  0.10  0.15 | <0.001  0.050  <0.001 | <0.001  0.229 | <0.001 |
|  |  |  |  |  |  |  |  |  |
| Val_1_ | 3a  WW | S2 in D all first minutes  = Post-transition  D1 (+11) + D2 (+31) + D3 (+51) + D4 (+61) | Unt  PTZ_7.5_  Val_1_  Val_1_+PTZ_7.5_ | 13.07  5.11  16.18  4.86 | 0.51  0.50  0.34  0.30 | <0.001  <0.001  <0.001 | <0.001  0.747 | <0.001 |
| Val_1_ | 3a  Whole well | S2 in L all first minutes  = Post-transition  L1 (+1) + L2 (+21) + L3 (+41) + L4 (+51) | Unt  PTZ_7.5_  Val_1_  Val_1_+PTZ_7.5_ | 3.27  20.47  5.34  20.10 | 0.37  0.62  0.30  0.50 | <0.001  0.040  <0.001 | <0.001  0.715 | <0.001 |
|  |  |  |  |  |  |  |  |  |
| Val_1_ | 3a  WW | S2 in D all non-first minutes  = Non-transition  D1 (12-20) + D2 (32-40) + D3 (52-60) + D4 (72-80) | Unt  PTZ_7.5_  Val_1_  Val_1_+PTZ_7.5_ | 9.93  9.32  14.97  9.51 | 0.17  0.24  0.13  0.15 | 0.564  < 0.001  0.645 | <0.001  0.838 | <0.001 |
| Val_1_ | 3a  WW | S2 in L all non-first minutes  = Non-transition  L1 (2-10) + L2 (22-30) + L3 (42-50) + L4 (62-70) | Unt  PTZ_7.5_  Val_1_  Val_1_+PTZ_7.5_ | 2.84  15.65  4.50  14.53 | 0.13  0.19  0.11  0.15 | <0.001  0.061  <0.001 | <0.001  0.205 | <0.001 |
|  |  |  |  |  |  |  |  |  |
| Val_2.5_ | 3a  WW | S2 in D (D in entire 4 cycles)  D1 (11-20) + D2 (31-40) + D3 (51-60) + D4 (71-80) | Unt  PTZ_7.5_  Val_2.5_  Val_2.5_+PTZ_7.5_ | 9.88  7.70  13.31  7.66 | 0.20  0.22  0.11  0.14 | 0.037  <0.001  0.014 | <0.001  0.969 | <0.001 |
| Val_2.5_ | 3a  WW | S2 in L (in all 4 entire cycles)  L1 (1-10) + L2 (21-30) + L3 (41-50) + L4 (61-70) | Unt  PTZ_7.5_  Val_2.5_  Val_2.5_+PTZ_7.5_ | 2.62  14.55  4.29  9.00 | 0.13  0.24  0.14  0.14 | <0.001  0.044  <0.001 | < 0.001  < 0.001 | <0.001 |
|  |  |  |  |  |  |  |  |  |
| Val_2.5_ | 3a  WW | S2 in D all first minutes  = Post-transition  D1 (+11) + D2 (+31) + D3 (+51) + D4 (+61) | Unt  PTZ_7.5_  Val_2.5_  Val_2.5_+PTZ_7.5_ | 13.34  5.16  15.38  11.34 | 0.58  0.47  0.33  0.51 | <0.001  0.074  0.080 | <0.001  <0.001 | <0.001 |
| Val_2.5_ | 3a  WW | S2 in L all first minutes  = Post-transition  L1 (+1) + L2 (+21) + L3 (+41) + L4 (+51) | Unt  PTZ_7.5_  Val_2.5_  Val_2.5_+PTZ_7.5_ | 3.38  20.29  5.32  12.85 | 0.42  0.80  0.30  0.54 | < 0.001  0.057  < 0.001 | < 0.001  < 0.001 | < 0.001 |
|  |  |  |  |  |  |  |  |  |
| Val_2.5_ | 3a  WW | S2 in D all non-first minutes  = Non-transition  D1 (12-20) + D2 (32-40) + D3 (52-60) + D4 (72-80) | Unt  PTZ_7.5_  Val_2.5_  Val_2.5_+PTZ_7.5_ | 9.50  7.98  13.08  7.26 | 0.20  0.24  0.12  0.14 | 0.161  <0.001  0.017 | <0.001  0.437 | <0.001 |
| Val_2.5_ | 3a  WW | S2 in L all non-first minutes  = Non-transition  L1 (2-10) + L2 (22-30) + L3 (42-50) + L4 (62-70) | Unt  PTZ_7.5_  Val_2.5_  Val_2.5_+PTZ_7.5_ | 2.54  13.91  4.17  8.57 | 0.13  0.24  0.11  0.14 | <0.001  0.055  <0.001 | <0.001  <0.001 | <0.001 |
|  |  |  |  |  |  |  |  |  |
| Val_5_ | 3a  WW | S2 in D (in all 4 entire cycles)  D1 (11-20) + D2 (31-40) + D3 (51-60) + D4 (71-80) | Unt  PTZ_7.5_  Val_5_  Val_5_+PTZ_7.5_ | 10.86  9.74  10.58  10.28 | 0.20  0.24  0.14  0.16 | 0.345  0.788  <0.001 | 0.411  0.597 | 0.720 |
| Val_5_ | 3a  WW | S2 in L (in all 4 entire cycles)  L1 (1-10) + L2 (21-30) + L3 (41-50) + L4 (61-70) | Unt  PTZ_7.5_  Val_5_  Val_5_+PTZ_7.5_ | 3.60  15.77  10.00  9.25 | 0.13  0.25  0.13  0.13 | <0.001  <0.001  <0.001 | <0.001  <0.001 | 0.286 |
|  |  |  |  |  |  |  |  |  |
| Val_5_ | 3c  WW | S2 in D all first minutes  = Post-transition  D1 (+11) + D2 (+31) + D3 (+51) + D4 (+61) | Unt  PTZ_7.5_  Val_5_  Val_5_+PTZ_7.5_ | 13.44  6.48  10.74  10.66 | 0.53  0.67  0.44  0.53 | <0.001  0.011  0.009 | <0.001  <0.001 | 0.929 |
| Val_5_ | 3c  WW | S2 in L all first minutes  = Post-transition  L1 (+1) + L2 (+21) + L3 (+41) + L4 (+51) | Unt  PTZ_7.5_  Val_5_  Val_5_+PTZ_7.5_ | 4.30  21.13  9.56  8.42 | 0.37  0.81  0.36  0.38 | <0.001  <0.001  <0.001 | <0.001  <0.001 | 0.072 |
|  |  |  |  |  |  |  |  |  |
| Val_5_ | 3e  WW | S2 in D all non-first minutes  = Non-transition  D1 (12-20) + D2 (32-40) + D3 (52-60) + D4 (72-80) | Unt  PTZ_7.5_  Val_5_  Val_5_+PTZ_7.5_ | 10.57  10.10  10.57  10.24 | 0.21  0.25  0.11  0.17 | 0.695  0.995  0.750 | 0.656  0.893 | 0.327 |
| Val_5_ | 3e  WW | S2 in L all non-first minutes  = Non-transition  L1 (2-10) + L2 (22-30) + L3 (42-50) + L4 (62-70) | Unt  PTZ_7.5_  Val_5_  Val_5_+PTZ_7.5_ | 3.52  15.18  10.05  9.343 | 0.14  0.26  0.13  0.14 | <0.001  <0.001  <0.001 | <0.001  <0.001 | <0.001 |
|  |  |  |  |  |  |  |  |  |
| Val_7_ | 3a  WW | S2 in D (in all 4 entire cycles)  D1 (11-20) + D2 (31-40) + D3 (51-60) + D4 (71-80) | Unt  PTZ_7.5_  Val_7_  VAL_7_+PTZ_7.5_ | 10.67  8.13  8.38  7.55 | 0.15  0.20  0.13  0.13 | 0.002  <0.001  <0.001 | 0.730  0.413 | 0.154 |
| Val_7_ | 3a  WW | S2 in L (in all 4 entire cycles)  L1 (1-10) + L2 (21-30) + L3 (41-50) + L4 (61-70) | Unt  PTZ_7.5_  Val_7_  VAL_7_+PTZ_7.5_ | 2.61  10.30  6.33  5.98 | 0.11  0.26  0.12  0.11 | <0.001  <0.001  <0.001 | <0.001  <0.001 | 0.533 |
|  |  |  |  |  |  |  |  |  |
| Val_7_ | 3a  WW | S2 in D all first minutes  = Post-transition  D1 (+11) + D2 (+31) + D3 (+51) + D4 (+61) | Unt  PTZ_7.5_  Val_7_  VAL_7_+PTZ_7.5_ | 12.90  7.18  9.45  9.09 | 0.42  0.51  0.44  0.50 | <0.001  <0.001  <0.001 | 0.006  0.021 | 0.593 |
| Val_7_ | 3a  WW | S2 in L all first minutes  = Post-transition  L1 (+1) + L2 (+21) + L3 (+41) + L4 (+51) | Unt  PTZ_7.5_  Val_7_  VAL_7_+PTZ_7.5_ | 3.14  14.10  6.40  6.85 | 0.37  0.93  0.32  0.34 | <0.001  <0.001  <0.001 | <0.001  <0.001 | 0.430 |
|  |  |  |  |  |  |  |  |  |
| Val_7_ | 3a  WW | S2 in D all non-first minutes  = Non-transition  D1 (12-20) + D2 (32-40) + D3 (52-60) + D4 (72-80) | Unt  PTZ_7.5_  Val_7_  VAL_7_+PTZ_7.5_ | 10.42  8.23  8.26  7.38 | 0.16  0.21  0.21  0.14 | 0.009  0.003  <0.001 | 0.976  0.234 | 0.135 |
| Val_7_ | 3a  WW | S2 in L all non-first minutes  = Non-transition  L1 (2-10) + L2 (22-30) + L3 (42-50) + L4 (62-70) | Unt  PTZ_7.5_  Val_7_  VAL_7_+PTZ_7.5_ | 2.55  9.88  6.32  5.88 | 0.12  0.26  0.13  0.12 | <0.001  <0.001  <0.001 | <0.001  <0.001 | 0.470 |
|  |  |  |  |  |  |  |  |  |
| Val_5_ | 3f  IS | S2 in D all first minutes  = Post-transition  D1 (+11) + D2 (+31) + D3 (+51) + D4 (+61) | Unt  PTZ_7.5_  Val_5_  Val_7_+PTZ_7.5_ | 5.51  1.36  1.42  2.60 | 0.31  0.21  0.11  0.16 | <0.001  <0.001  <0.001 | 0.895  0.003 | <0.001 |
| Val_5_ | 3f  IS | S2 in L all first minutes  = Post-transition  L1 (+1) + L2 (+21) + L3 (+41) + L4 (+51) | Unt  PTZ_7.5_  Val_5_  Val_7_+PTZ_7.5_ | 0.73  3.62  0.99  1.51 | 0.12  0.34  0.07  0.10 | <0.001  0.328  0.003 | <0.001  <0.001 | 0.017 |
|  |  |  |  |  |  |  |  |  |
| Val_5_ | 3g  IS | S3 in D all first minutes  = Post-transition  D1 (+11) + D2 (+31) + D3 (+51) + D4 (+61) | Unt  PTZ_7.5_  Val_5_  Val_7_+PTZ_7.5_ | 0.13  0.36  0.22  0.43 | 0.03  0.08  0.02  0.04 | 0.002  0.188  <0.001 | 0.028  0.322 | <0.001 |
| Val_5_ | 3g  IS | S3 in L all first minutes  = Post-transition  L1 (+1) + L2 (+21) + L3 (+41) + L4 (+51) | Unt  PTZ_7.5_  Val_5_  Val_7_+PTZ_7.5_ | 0.01  1.96  0.27  0.57 | 0.00  0.04  0.01  0.01 | <0.001  0.049  <0.001 | <0.001  <0.001 | 0.004 |
