# Supplementary material for: Reversal of pentylenetetrazole-altered swimming and neural activity-regulated gene expression in zebrafish larvae by valproic acid and valerian extract
Source: Psychopharmacology (Berl). 2016 May 11;233:2533–47. doi: 10.1007/s00213-016-4304-z (PMC4908174; doi:10.1007/s00213-016-4304-z)
Supplement: Supplementary file 7 — (DOCX 24 kb) [file 213_2016_4304_MOESM7_ESM.docx]

**Table 7** STATA analyses of distances traveled in swim speed S2 by untreated (Unt) vs. (PTZ_7.5_, VPA_2_, VPA_2_+ PTZ_7.5_-treated) larvae during all successive transitions (Fig.3b)

**Note**: We used a modified Brown and Forysthe test giving results in the format of a 95% Confidence Intervals (CI). When 0 (zero) is not included in the IC the result is considered significant

| **Fig.3b**  **all transitions**  **whole plate**  **(WW)**  **in S2** | **Treatment** | **Mean** | **SEM** | **p value**  **Ref Unt** | **p value**  **Ref PTZ_7.5_** | **p value**  **Ref Val_5_** |
| --- | --- | --- | --- | --- | --- | --- |
| L1 (min1) | Unt  PTZ_7.5_  Val_5_  Val_5_ +PTZ_7.5_ | 4.83  15.53  10.20  9.52 | 0.64  1.63  0.69  0.77 | -15.89 – -5.506  -8.090 – -2.660  -7.587 – -1.804 | 0.0910 – 10.560  0.6880 – 11.320 | -2.284 – 3.643 |
| D1 (min11) | Unt  PTZ_7.5_  Val_5_  Val_5_+PTZ_7.5_ | 12.80  8.58  12.65  13.67 | 1.39  1.78  0.83  1.00 | -2.356 – 10.801  -4.583 – 4.887  -5.852 – 4.115 | -9.870 – 1.720  -11.09 – 0.898 | -4.746 – 2.704 |
| L2 (min21) | Unt  PTZ_7.5_  Val_5_  Val_5_+PTZ_7.5_ | 3.16  20.99  10.46  8.88 | 0.58  1.37  0.68  0.70 | -22.24 – -13.42  -9.878 – -4.721  -8.331 – -3.098 | 6.018 – 15.04  7.584 – 16.65 | -1.211 – 4.381 |
| D2 (min31) | Unt  PTZ_7.5_  Val_5_  Val _5_+PTZ_7.5_ | 13.70  5.32  10.92  11.58 | 1.06  1.02  0.86  1.26 | 4.095 – 12.672  -1.180 – 6.743  -2.139 – 6.376 | -9.470 – -1.740  -10.43 – -2.10 | -4.484 – 3.159 |
| L3 (min41) | Unt  PTZ_7.5_  Val_5_  Val_5_+PTZ_7.5_ | 4.05  24.25  9.04  7.76 | 0.69  1.40  0.73  0.78 | -24.80 – -15.59  -7.877 – 2.098  -6.669 – -0.739 | 10.560 – 19.850  11.800 – 21.180 | -1.739 – 4.306 |
| D3 (min51) | Unt  PTZ_7.5_  Val_5_  Val _5_+PTZ_7.5_ | 13.70  4.97  10.05  9.29 | 0.93  1.27  0.90  1.08 | 4.125 – 13.332  -0.094 – 7.390  0.313 – 8.499 | -9.629 – -0.534  -9.152 – 0.506 | -3.264 – 4.050 |
| L4 (min61) | Unt  PTZ_7.5_  Val_5_  Val_5_+PTZ_7.5_ | 5.15  23.74  8.52  7.29 | 0.94  1.50  0.72  0.77 | -23.77 – -13.41  -6.828 – 0.082  -5.890 – 1.177 | 10.315 – 20.123  11.280 – 21.191 | -2.017 – 4.050 |
| D4 (min71) | Unt  PTZ_7.5_  Val_5_  Val_5_+PTZ_7.5_ | 13.55  7.05  9.33  8.10 | 0.86  1.09  0.85  0.96 | 2.44 – 10.57  0.726 – 7.729  1.743 – 9.174 | -6.307 – 1.753  -5.257 – 3.166 | -2.438 – 4.900 |
