# Supplementary material for: Reversal of pentylenetetrazole-altered swimming and neural activity-regulated gene expression in zebrafish larvae by valproic acid and valerian extract
Source: Psychopharmacology (Berl). 2016 May 11;233:2533–47. doi: 10.1007/s00213-016-4304-z (PMC4908174; doi:10.1007/s00213-016-4304-z)
Supplement: Supplementary file 8 — (DOCX 24 kb) [file 213_2016_4304_MOESM8_ESM.docx]

**Table 8** STATA analyses of inner distances traveled in swim speed S2 by untreated (Unt) versus (PTZ_7.5_, Val_5_, PTZ_7.5_+Val_5_-treated) larvae during all successive transitions (Fig.3h)

**Note**: We used a modified Brown and Forysthe test giving results in the format of a 95% Confidence Intervals (CI). When 0 (zero) is not included in the IC the result is considered significant

| **Fig.3h**  **all transitions**  **inner space**  **(IS)**  **in S2** | **Treatment** | **Mean** | **SEM** | **95% CI**  **Ref Unt** | **95% CI**  **Ref PTZ** | **95% CI**  **Ref Val_5_** |
| --- | --- | --- | --- | --- | --- | --- |
| L1 (min1) | Unt  PTZ_7.5_  Val_5_  Va_l5_ +PTZ_7.5_ | 0.73  1.78  1.18  1.59 | 0.19  0.22  0.16  0.22 | -1.924 – -0.184  -1.188 – 0.292  -1.712 – -0.004 | -0.216 – 1.423  -0.729 – 1.121 | -1.215– 0.394 |
| D1 (min11) | Unt  PTZ_7.5_  Val_5_  Val_5_+PTZ_7.5_ | 4.68  1.71  2.29  3.34 | 0.70  0.53  0.24  0.34 | 0.406– 5.528  0.183 – 4.596  -0.962 – 3.637 | -2.292 – 1.138  -3468 – 0.210 | -2.251 – 0.147 |
| L2 (min21) | Unt  PTZ_7.5_  Val_5_  Val_5_+PTZ_7.5_ | 0.81  2.82  1.22  1.62 | 0.34  0.41  0.16  0.19 | -3.562 – -0.446  -1.534 – 0.713  -1.965 – 0.352 | 0.280 – 2.907  -0.145 – 2.54 | -1.133 – 0.342 |
| D2 (min31) | Unt  PTZ_7.5_  Val_5_  Val _5_+PTZ_7.5_ | 5.74  1.29  1.43  2.83 | 0.57  0.45  0.20  0.30 | 2.313 – 6.579  2.498 – 6.123  1.003 – 4.817 | -1.609 – 1.338  -3.128 – 0.057 | -2.450 – -0.350 |
| L3 (min41) | Unt  PTZ_7.5_  Val_5_  Val _5_+PTZ_7.5_ | 0.72  4.45  0.89  1.59 | 0.24  0.74  0.11  0.22 | -6.034 – -1.407  -0.940 – 0.620  -1.815 – 0.082 | 1.312 – 5.801  0.551 – 5.158 | -1.431 – 0.019 |
| D3 (min51) | Unt  PTZ_7.5_  Val_5_  Val_5_+PTZ_7.5_ | 5.53  0.90  1.05  2.07 | 0.54  0.33  0.16  0.27 | 2.753 – 6.505  2.782 – 6.185  1.666 – 5.259 | -1.238 – 0.947  -2.412 – 0.079 | -1.928 – -0.113 |
| L4 (min61) | Unt  PTZ_7.5_  Val_5_  Val_5_+PTZ_7.5_ | 0.65  5.42  0.67  1.24 | 0.20  0.91  0.10  0.17 | -7.588 – -1.962  -0.691 – 0.649  -1.356 – 0.177 | 1.976 – 7.531  1.387 – 6.984 | -1.138 – 0.001 |
| D4 (min71) | Unt  PTZ_7.5_  Val_5_  Val_5_+PTZ_7.5_ | 6.07  1.52  0.88  2.12 | 0.63  0.34  0.17  0.29 | 2.417 – 6.682  3.218 – 7.165  1.872 – 6.019 | -0.502 – 1.785  -1.927 – 0.719 | -2.236 – -0.256 |
