# Supplementary material for: Reversal of pentylenetetrazole-altered swimming and neural activity-regulated gene expression in zebrafish larvae by valproic acid and valerian extract
Source: Psychopharmacology (Berl). 2016 May 11;233:2533–47. doi: 10.1007/s00213-016-4304-z (PMC4908174; doi:10.1007/s00213-016-4304-z)
Supplement: Supplementary file 9 — (DOCX 24 kb) [file 213_2016_4304_MOESM9_ESM.docx]

**Table 9** STATA analyses of inner distances traveled in swim speed S3 by untreated (Unt) versus (PTZ_7.5_, Val_5_, PTZ_7.5_+Val_5_-treated) larvae during all successive transitions (Fig.3h)

**Note**: We used a modified Brown and Forysthe test giving results in the format of a 95% Confidence Intervals, which when not including 0 are considered significant

| **Fig.3h**  **all transitions**  **inner space**  **(IS)**  **in S3** | **Treatment** | **Mean** | **SEM** | **95% CI**  **Ref Unt** | **95% CI**  **Ref PTZ** | **95% CI**  **Ref Val_5_** |
| --- | --- | --- | --- | --- | --- | --- |
| L1 (min1) | Unt  PTZ_7.5_  Val _5_  Val _5_ +PTZ_7.5_ | 0.00  1.35  0.29  0.70 | 0.00  0.23  0.05  0.13 | -2.117 – -0.742  -0.453 – -0.118  -1.109 – -0.316 | 0.440 – 1.847  -0.060 – 1.492 | -0.854 – 0.000 |
| D1 (min11) | Unt  PTZ_7.5_  Val _5_  Val _5_+PTZ_7.5_ | 0.10  0.34  0.29  0.61 | 0.03  0.10  0.05  0.09 | -0.538 – 0.096  -0.416 – -0.502  -0.800 – -0.200 | -0.348 – 0.323  -0.684 – 0.126 | -0.587 – 0.054 |
| L2 (min21) | Unt  PTZ_7.5_  Val _5_  Val _5_+PTZ_7.5_ | 0.00  1.75  0.33  0.64 | 0.00  0.31  0.06  0.09 | -2.729 – -0.863  -0.513 – -0.137  -0.926 – -0. 370 | 0.523 – 2.418  0.183 – 2.112 | -0.653 – 0.007 |
| D2 (min31) | Unt  PTZ_7.5_  Val _5_  Val _5_+PTZ_7.5_ | 0.24  0.16  0.23  0.40 | 0.10  0.05  0.05  0.07 | -0.261 – 0.443  -0.342 – 0.359  -0.531 – 0.226 | -0.293 – 0.126  -0.502 – 0.014 | -0.415 – 0.094 |
| L3 (min41) | Unt  PTZ_7.5_  Val _5_  Val _5_+PTZ_7.5_ | 0.02  2.21  0.27  0.47 | 0. 02  0.31  0.05  0.09 | -3.166 – -1.300  -0.428 – -0.076  -0.740 – -0.173 | 1.038 – 2.923  0.814 – 2.740 | -0.524 – 0.115 |
| D3 (min51) | Unt  PTZ_7.5_  Val _5_  Val _5_+PTZ_7.5_ | 0.07  0.50  0.17  0.37 | 0.02  0.28  0.04  0.08 | -1.286 – 0.428  -0.246 – 0.051  -0.542 – -0.045 | -0.531 – 1.193  -0.745 – 1.015 | -0.464 – 0.073 |
| L4 (min61) | Unt  PTZ_7.5_  Val _5_  Val_5_+PTZ_7.5_ | 0.02  2.20  0.17  0.43 | 0.02  0.26  0.05  0.06 | -3.090 – -1.578  -0.312 – 0.004  -0.600 – -0.195 | 1.414 – 2.944  1.162 – 2.708 | -0.483 – 0.004 |
| D4 (min71) | Unt  PTZ_7.5_  Val_5_  Val_5_+PTZ_7.5_ | 0.10  0.48  0.10  0.34 | 0.06  0.14  0.02  0.06 | -0.821 – 0.104  -0.234 – 0.191  -0.497 – 0.038 | -0.100 – 0.776  -0.335 – 0.594 | -0.4222 – 0.006 |
